# Cationic Antimicrobial Polymers and Their Assemblies

**DOI:** 10.3390/ijms14059906

**Published:** 2013-05-10

**Authors:** Ana Maria Carmona-Ribeiro, Letícia Dias de Melo Carrasco

**Affiliations:** 1 Biocolloids Lab, Departamento de Bioquímica, Instituto de Química, Universidade de São Paulo, Caixa Postal 26077-05513-970, São Paulo, Brazil; E-Mail: lemelodias@usp.br; 2 Departamento de Análises Clínicas e Toxicológicas, Faculdade de Ciências Farmacêuticas, Universidade de São Paulo, CEP 05508-900, São Paulo, Brazil

**Keywords:** cationic polymers, cationic surfactants and lipids, self-assembled films and nanoparticles, antimicrobial activity

## Abstract

Cationic compounds are promising candidates for development of antimicrobial agents. Positive charges attached to surfaces, particles, polymers, peptides or bilayers have been used as antimicrobial agents by themselves or in sophisticated formulations. The main positively charged moieties in these natural or synthetic structures are quaternary ammonium groups, resulting in quaternary ammonium compounds (QACs). The advantage of amphiphilic cationic polymers when compared to small amphiphilic molecules is their enhanced microbicidal activity. Besides, many of these polymeric structures also show low toxicity to human cells; a major requirement for biomedical applications. Determination of the specific elements in polymers, which affect their antimicrobial activity, has been previously difficult due to broad molecular weight distributions and random sequences characteristic of radical polymerization. With the advances in polymerization control, selection of well defined polymers and structures are allowing greater insight into their structure-antimicrobial activity relationship. On the other hand, antimicrobial polymers grafted or self-assembled to inert or non inert vehicles can yield hybrid antimicrobial nanostructures or films, which can act as antimicrobials by themselves or deliver bioactive molecules for a variety of applications, such as wound dressing, photodynamic antimicrobial therapy, food packing and preservation and antifouling applications.

## 1. Introduction

Antibiotics are naturally occurring or synthetic organic compounds which inhibit or destroy bacteria or other microorganisms, generally at low concentrations. Antiseptics are biocides or products that destroy or inhibit the growth of microorganisms in or on living tissue (e.g., health care personnel handwashes and surgical scrubs); and disinfectants are similar but generally are products or biocides that are used on inanimate objects or surfaces [1]. Antiseptics and disinfectants are used extensively in hospitals and other health care settings. They are an essential part of infection control practices and prevention of nosocomial infections [2,3]. The potential for microbial contamination and infection risks in the food and general consumer markets have also led to increased use of antiseptics and disinfectants in general. Active biocidal chemicals are found in these products, many of which have been used for hundreds of years for antisepsis, disinfection, and preservation [4].

Antibiotic-resistant bacteria are an important threat to public health due to the slow development of new antibiotics to replace those that become ineffective. Novel antimicrobial agents are needed and much work has been devoted to developing highly efficient compounds that are also less susceptible to development of resistance by the bacteria. Cationic compounds have emerged as promising candidates for further developments as antimicrobial agents with decreased potential for resistance development. Among them, cationic surfactants, lipids, peptides and natural or synthetic polymers have been intensively searched as antimicrobial agents by themselves or in sophisticated formulations.

Quaternary ammonium compounds (QACs) are the most useful antiseptics and disinfectants [5,6]. QACs have been used for a variety of clinical purposes (e.g., preoperative disinfection of unbroken skin, application to mucous membranes, and disinfection of noncritical surfaces). In addition to having antimicrobial properties, QACs are also excellent for hard-surface cleaning and deodorization. QACs are membrane active agents [7] (*i.e.*, with a target site predominantly at the cytoplasmic (inner) membrane in bacteria or the plasma membrane in yeasts) [8]. The following sequence of events occurs with microorganisms exposed to cationic agents: (i) adsorption and penetration of the agent into the cell wall; (ii) reaction with the cytoplasmic membrane (lipid or protein) followed by membrane disorganization; (iii) leakage of intracellular low-molecular-weight material; (iv) degradation of proteins and nucleic acids; and (v) wall lysis caused by autolytic enzymes. There would be a loss of structural organization and integrity of the cytoplasmic membrane in bacteria, together with other damaging effects to the bacterial cell [9,10]. Actually, most studies on membrane damage evaluated the effects of biocides on protoplasts and spheroplasts suspended in various solutes. QACs caused lysis of spheroplasts and protoplasts suspended in sucrose [8,11–13]. The cationic agents interact with phospholipid components in the cytoplasmic membrane [14], thereby producing membrane distortion and protoplast lysis under osmotic stress. Isolated membranes, however, did not undergo disaggregation on exposure to QACs. Some polymeric quaternary ammonium compounds such as polyquaternium-1, a quaternary ammonium polymeric compound, and myristamidopropyl dimethylamine, were shown to induce lysis of spheroplasts of *S. marcescens*, but not those of *C. albicans* [15]. Damage to the membrane was sufficient to cause K^+^ leakage but this injury was not always sufficient to cause spheroplasts lysis. Adsorption of dioctadecyldimethyl ammonium bromide (DODAB) cationic bilayers onto bacterial cells changed the sign of the cell surface potential from negative to positive and a clear relationship between positive charge on bacterial cells and death was described [16]. Regarding the mechanism of DODAB action, neither bacterial cell lysis nor DODAB vesicle disruption took place [17] in contrast to the mechanism of action for single chained cationic surfactants [8,11–13].

The deposition of organic monolayers containing quaternary ammonium groups has been shown by many authors to confer biocidal properties on a large variety of solid surfaces. Quaternized poly (vinylpyridine) chains were grafted on glass surfaces and the charge density varied within the organic layer between 10^12^ and 10^16^ positive charges per cm^2^ so that the effect of the surface charge density on the biocidal activity could be determined [18]. There is a charge-density threshold for optimal efficiency of biocidal action preventing deposition of bacterial biofilms [18]. The removal of divalent counterions from the bacteria during adsorption on charged surfaces might have induced disruption of the bacterial envelope and loss of cell viability.

Although it is quite well known that cationic molecules in solution are able to kill bacteria [19–22], only recently positive charges attached to surfaces, particles, polymers, liposomes or bilayers have really been used to kill bacteria upon contact [23–30]. Positively charged moieties were the quaternary ammonium [28] or the phosphonium [29,30]. Various cationic architectures have been tested such as the polyelectrolyte layers [28,30–32] and the dendrimers [33–35].

In this review, cationic antimicrobial agents based on polymers, lipids and their assemblies are discussed. Table 1 shows the molecular or supramolecular structure of some cationic surfactants, lipids, polymers and their assemblies reported to exhibit potent antimicrobial activity both by themselves or in combinations with inert materials such as natural polymers, eg carboxymethylcellulose (CMC) or even synthetic polymers such as poly (acrylates) or polystyrene sulfate in form of microspheres. Table 1 also illustrates the wide range of cationic molecular and supramolecular structures including nanoparticles that displayed antimicrobial properties. Besides antibiotics and antiviral drugs, a wide range of surfactants, lipids, polymers, nanoparticles and their assemblies can act as antimicrobials.

## 2. Cationic Antimicrobial Surfactants, Lipids and Polymers

Both cationic surfactants and polymers with the quaternary ammonium moiety in their chemical structures find many applications in conditioners, shampoo, hair mousse, hair spray, hair dye, and contact lens solutions. Because they are positively charged, they neutralize the negative charges of most shampoos and hair proteins helping the hair to lie flat. Their positive charges also ionically bind them to hair and skin. Some of them have antimicrobial properties. For example, potential targets for polyquartenium-1 (PQ-1), a much-studied cationic polymer, are the cytoplasmic membrane of the bacteria and the plasma membrane of the fungi, since these are common targets for QACs [1]. K^+^ leakage is an ideal indicator of membrane damage as it leaks out of the cells very rapidly, and can be easily detected by atomic absorption spectroscopy [48]. There are large differences between the organisms regarding the amount of K^+^ released after treatment with PQ-1 due to intrinsic differences between the organisms. For example, an amoeba has a very different physiology from a bacterial cell and it would thus be expected that the different types of organism contain different levels of potassium. This was indeed observed in different cells lysed by boiling [15]. PQ-1 induced K^+^ leakage with possible membrane damage to *Aspergillus fumigatus* and *Candida albicans* but potassium leakage was absent from *Acanthamoeba castellanii*. This was probably due to the presence of a cell wall, especially in the cyst form of the organism that is very resistant to damage [15].

Quaternary ammonium salts (quats) were obtained for the first time from the irreversible and very efficient reaction between tertiary amines and alkyl halides [49]. Applications for quats rapidly developed during the 20th century. Their bactericidal activity was described [49–53] but applications for disinfection were only reported in 1935 [54]. In the 1960s, the compounds were found to be excellent fabric softeners. In 1965, their important role in phase transfer catalysis was shown [55,56]. In the 70s the compounds were used as antielectrostatic agents and wood preservatives [57,58]. In the 80s, they were used as asphalt modifiers and in the nineties as clay modifiers. At the end of 20th century, quats with certain counterions such as BF^4−^, PF^6−^, (CF_3_SO_2_)_2_N^−^ and others were named ionic liquids [59–65]. Briefly, their history points to several important industrial applications. They consist of an organic cation and an inorganic or organic anion. The most important disadvantages of quats applications are resistance in microbes [66] and the relatively slow decomposition in the natural environment due to their high chemical stability [67,68]. Several interesting polymeric [69–73] or polymerizable [74–79] cationic amphiphiles have been synthesized in recent years, some of them with the major objective of gene delivery [72,80–82] and a few aiming at evaluation of antimicrobial or other biological effects [83–86]. DNA/polycation complexes formed by polycations with quaternary amine groups, *i.e.*, poly(*N*-alkyl-4-vinylpyridinium) bromides, poly(*N,N*-dimethyldiallylammonium) chloride, and ionene bromide, were pH independent and the least tolerant to dissociation by the added salt [81]. Primary amine groups of basic polypeptides poly-l-lysine hydrobromide and poly-l-arginine hydrochloride as well as synthetic polycation poly(vinyl-2-aminoethyl ether) provided the best stability to DNA/polycation complexes in water-salt solutions under a wide pH range [81]. Moderate and pH-dependent stability was revealed for DNA complexes formed with poly(*N*,*N*-dimethylaminoethylmethacrylate) with tertiary amine groups in the chain or branched poly(ethylenimine) with primary, secondary, and tertiary amine groups in the molecule. Quaternization of a part of tertiary amine groups of poly(*N*,*N*-dimethylaminoethylmethacrylate) resulted in reduction of the stability of the DNA-containing complexes in water-salt solutions. The dissociation of complexes formed by random copolymer of 4-vinylpyridine and *N*-ethyl-4-vinylpyridinium bromide was pH sensitive and could be performed under pH and ionic strength close to the physiological conditions [81].

**Figure 1:**
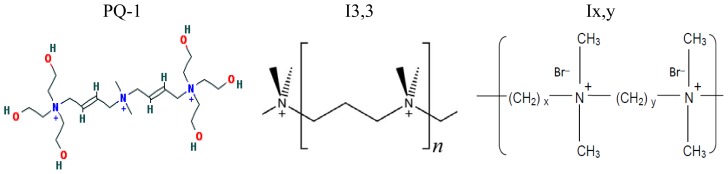
Polyquaternium-1 (PQ-1) (adapted from [15]), 3,3-ionene (I3,3) and x,y-ionene (Ix,y) (adapted with permission from [87]).

Ionenes are among the first synthesized cationic polymers with quaternary ammonium moieties in their chains separated by hydrophobic moieties. The first report on ionenes dates back to the 1930s [84]. In the 1940s, kinetics and thermodynamics of ionenes formation was extensively studied [83]. Polyionenes biological effects were: (1) bactericidal action, (2) formation of insoluble complexes with DNA and heparin, (3) neuromuscular blocking action, (4) cell aggregation and lysis, and (5) cell adhesion [87]. In 1977, the interaction of living cells with polyionenes and polyionene-coated surfaces was studied [87]. The I3,3 and I6,10 structures were used as molecular probes to gain an understanding of the cell surface phenomena of adhesion on glass- and on polyionenes-treated surfaces. A study of the interaction of polyionenes in solution *in vitro* and *in vivo* and polyionenes covalently bound to polymeric microspheres with leukemic murine EL4 cells and normal thymocytes showed specific cytotoxity towards the leukemic cells. Figure 1 shows the chemical structures of polyquaternium-1 and two ionenes.

Hexadimethrine bromide, 3,6-ionene, with different molecular weights (MW) were isolated allowing the determination of the effect of MW on the LD50 *in vivo*: LD50 decreases with increasing ionene chain length [88]. The effective fractionation of polydisperse samples of ionenes allowed to obtain samples of 2,4-, and 2,8-ionenes in the 10 to 30 range of polymerization degree, DP [85]. The cytotoxicity of the ionenes against P388D1 macrophages was low with the minimum viability of the cells at various concentrations of polycation at 80% relative to controls. In comparison with polyethyleneimine and polylysine, ionenes exhibit a low cytotoxicity, a truly essential requirement for cell-based transfection assays [89]. The cytotoxicity depends on polycation nature (primary, secondary, tertiary, or quaternary amine group) rather than the macromolecule DP or its charge density. Polymeric quaternary ammonium salts possessed little activity as gene delivery agents. While most of the polycations with different types of charged moieties are able to condense DNA into small particles with an overall positive surface charge, the transfection efficiency dramatically drops from polylysine-based vectors to quaternary ammonium polymers [90].

A strong interaction was observed between the polyionenes and acidic phospholipidic bilayers whereas zwitterionic phospholipid bilayers were not affected by the polycations [86]. Polyionenes with rigid spacers were found to be most effective to induce phase separation in model phospholipid bilayer vesicles and the most active as antimicrobial agents [86]. Polyionenes with alternate rigid and flexible spacers exhibited antimicrobial activities similar to those with flexible spacers only. The rigid spacers are favorable for strong interaction with membranes which are assumed to be the target sites of the polycationic biocides implicated in a high antimicrobial activity [86]. Other factors affecting both the antimicrobial activity and the mode of interaction with membranes were molecular weight and hydrophobicity. With increasing molecular weight, both the activity and ability to induce phase separation increased. Introduction of hydrophilic groups into the spacers resulted in loss of activity and ability to induce phase separation [86].

**Figure 2:**
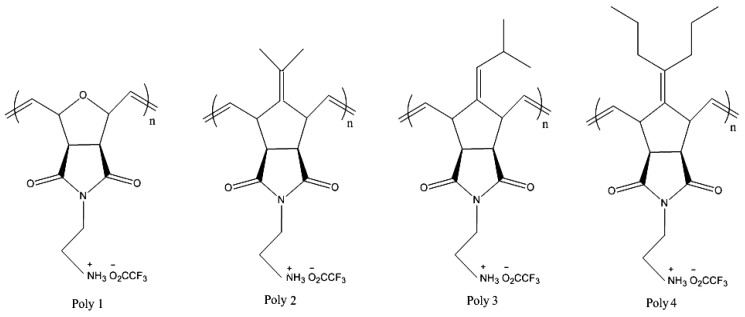
Amphiphilic polynorbornene derivatives with variable hydrophobic moieties in the repeating unities. Adapted with permission from [92].

Diels-Alder cycloaddition of fulvene derivatives and dienophiles allowed the preparation of 2,3-disubstituted-7-alkylidene norborn-2-ene derivatives (1–4) [91]. Depending on the monomer and the catalyst, water-soluble amphiphilic polymers over a range of molecular weights and narrow polydispersity indexes (1.08–1.20) were quantitatively obtained [91]. To probe the effect of polymer molecular weight, ionic nature, and hydrophobic character on membrane disruption activity, polymer-induced dye leakage from large unilamellar vesicles was measured. The presence, and balance, of a hydrophobic group and a cationic group were critical to achieve high activities. The membrane disruption activity of cationic amphiphilic polymers was found to reach a maximum at a critical molecular weight [91]. Later on, the hemolytic and antibacterial activities for a series of cationic polynorbornene derivatives (Figure 2) showed that the hydrophobicity of the repeat unity dramatically affected antibacterial and hemolytic activities [92]. By tuning the overall hydrophobicity of the polymer through random copolymerizations of modular norbornene derivatives, highly selective, nonhemolytic antibacterial activities were obtained [92]. The overall efficacy toward both Gram-negative and Gram-positive bacteria was strongly dependent on the length of alkyl substituents on the repeat units. The activity of each homopolymer with similar molecular weights (near 10,000 g/mol, Mn) was probed against Gram-negative bacteria (*E. coli*), Gram-positive bacteria (*B. subtilis*), and human red blood cells [92].

Poly1, a cationic polymer with no substantial hydrophobic group, did not show any antibacterial or hemolytic activity in accordance with the lack of activity against phospholipid membranes [91]. Introduction of a hydrophobic group at the repeat unit level produced an increase in antibacterial and hemolytic activities, which depended on the size of hydrophobic group. Poly2, with an isopropylidene pendant group, exhibited antibacterial activity with an MIC of 200 μg/mL against *E. coli*, which is less efficacious than most antimicrobial cationic surfactants. However, poly2 remained nonhemolytic up to the measured concentration of 4,000 μg/mL. Poly3 exhibited a substantial increase in antibacterial activity (MIC = 25 μg/mL for both *Escherichia coli* and *Bacillus subtilis*) and in hemolytic activity, an HC50 of less than 1 μg/mL. For poly4, the polymer with the largest hydrophobic moiety, the hemolytic activity was retained, but the antibacterial activity decreased (MIC = 200 μg/mL). Electrostatic interactions were once again suggested to be more important for antibacterial activity [92].

**Figure 3:**
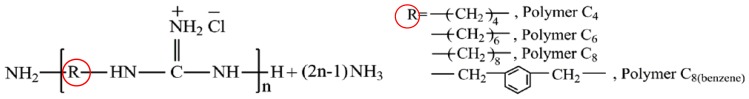
Polyhexamethylene biguanide (PHMB) and its derivatives. Adapted with permission from [106].

Polyhexamethylene biguanide (PHMB) is a broadspectrum antibacterial compound that also acts as a biocide against some fungi and protozoa and has been widely used for many years as an antiseptic in medicine. Its current applications also include swimming pool sanitisation, the treatment of cooling systems to prevent infection by *Legionella* [93], solid surface cleaners in the food industry, as a treatment against fungi [94] and *Acanthamoeba* [95–97] in infective keratitis, as a disinfecting contact lens solution [98], as an antibacterial mouth rinse [99,100], as a durable anti-odour finish in textiles [101], impregnation of gauze wound-dressing to prevent *Pseudomonas* infection [102], and the treatment of hatching eggs to prevent *Salmonella* infection [103,104]. Preparations of PHMB are mixtures of polymeric biguanides with a molecular weight range of 400–8,000 representing polymers with 2–40 repeating unities, respectively, and an average size at n = 11. PHMB polymers interact strongly and cooperatively with nucleic acids *in vitro* and this was related to the mechanism of the PHMB bactericidal action [105]. PHMB has been considered a promising candidate to further quantitative structure-activity relationship (QSAR) studies. Recently, analogs of PHMB with increasing hydrocarbon chain lengths in the repeating unity were synthesized and their activity against antibiotics-resistant clinically isolated strains was evaluated [106]. The gradually increased overall hydrophobicity with the increasing alkyl chain length in Polymers C_4_ to C_8_ lead to better partition in the hydrophobic regions of the phospholipids membrane, further causing stronger binding (higher binding constant and higher heat effects values) to the membrane, which lastly resulted in the gradually increased antimicrobial ability [106]. The rigid benzene ring of Polymer C8 (benzene) limited its partition into the lipid membrane and led to its weaker membrane-disrupting ability compared to the flexible alkyl chain of Polymer C8. These compounds were tested on 370 clinical strains and exhibited broad bactericidal and fungicidal activities against both antibiotics-susceptible and -resistant strains with a MIC range of 1–64 mg/L, being especially efficient against methicillin-susceptible and -resistant *Staphylococcus aureus* and coagulase-negative *Staphylococci* [106]. The chemical structure of PHMB and analogs is shown in Figure 3.

In summary, the membrane-disrupting ability of amphiphilic cationic polymers has been used for preparing disinfectants and biocides [107]. This disruption mechanism is enhanced by the cooperative action inherent in polymeric structures as compared to small amphiphilic molecules, such as surfactants [108]. Important features of polymers useful as disinfectants are not only their antimicrobial activity but also the absence of toxicity to human cells, particularly for medical and clinical utility. Therefore, differential cytotoxicity is a central issue that should always be addressed in the evaluation of cationic amphiphilic polymers for further applications *in vivo*. For the cationic lipid dioctadecyldimethylammonium bromide (DODAB), data on its differential toxicity against several mammalian cells lines including transformed cancer cells are shown in Table 2. This set of data suggests that DODAB can selectively kill the pathogenic microorganisms at least *in vitro.*

Both cationic lipids and polymers are very actively investigated for gene therapy applications [111]. *In vivo* gene administration into the patient or into the target organ requires formulation of the gene to avoid clivage by DNAases and other hurdles that occur *in vivo* such as complement activation. The *ex vivo* administration includes harvesting and cultivation of cells from patients, with *in vitro* gene transfer and reintroduction of transfected cells. The potential target cells for this administration include lymphocytes, bone marrow cells, umbilical cord blood stem cells, hepatocytes, tumor cells, and skin fibroblasts [111]. The main challenges of gene therapy both *ex vivo* and *in vivo*, are the delivery of DNA to target cells accompanied with a high, and permanent level of the desired gene expression. While lipids that facilitate transformation of lipoplexes to non-bilayer phases mediate high transfection activity *in vitro*, lipids, like cholesterol, that confer stability in serum, are more suitable for gene delivery *in vivo* [112]. The efficiency of polyplex-mediated transfection depends on the ability of the polymer to condense DNA, while allowing it to dissociate once inside the cell [112]. Novel cationic lipids and polymers aiming at gene therapy remain open to further studies in order to establish their activity as suitable anti-infective agents. Basically, gene therapy applications rely on the formation of DNA/cationic lipid or DNA/cationic polymer complexes via electrostatic interactions between the cationic moiety in the lipid or the polymer and the negatively charged DNA. Although their structure/activity relationships are not well understood, it is generally agreed that the nature of the positive headgroup impacts on their transfection activity. Several outstanding review articles appeared focusing on the relationship between the structure of the cationic lipid and the transfection activity and the main structure/activity findings have been reviewed [113–118]. Beautiful and novel molecular architectures have appeared that represent a real promise also as antibacterial or antiinfective agents [119–127], though they were not investigated from this point of view. Studies with BGBH (bis-guanidinium bis(2-aminoethyl)amine hydrazine) polar headgroup [128], guanidinylated polymerizable diacetylene lipids [129], aminoglycoside-derived lipids [117] and other cationic lipids suggested in general that the decrease in transfection activity at high charge ratio might reflect toxic effects of the cationic lipid [130]. For the delivery of DNA by polyplexes prepared from poly (2-dimethylaminoethyl methacrylate) (PDMAEMA) the transfection efficiency dramatically increased with the polymer molecular weight but the cellular uptake of polyplexes was determined to be insensitive to PDMAEMA molecular weight and there was no correlation between polyplex size and transfection efficiency [131]. The intracellular fate of the polyplexes, which involves endosomal release and DNase resistance, is more important to overall transfection efficiency than barriers to entry, such as polyplex size [131].

Determination of the specific elements in polymers, which affect their biological activity, has previously been difficult due to the broad molecular weight distributions and random sequences characteristic of radical polymerization. With advances in the polymerization control [132–134], well-defined polymers and selection of amphiphilic block or alternating polymer structures are allowing greater insight into their antimicrobial mechanism. For example, pegylated-polymers with tertiary amines from readily available commodity monomers 2-(dimethylamino)ethyl methacrylate (DMAEMA) and oligo (ethylene glycol) Me ether methacrylate (OEGMA, Mn ~475 Da) were synthesized by reversible addition-fragmentation chain transfer (RAFT) polymerization [133]. By employing a simple and efficient post-polymerization functionalization strategy, tertiary amines were quaternized to produce antimicrobial cationic polymers. Antimicrobial and hemolytic activities of amphiphilic polymethacrylate derivatives were tailored by alternating the content of hydrophobic groups and molecular weights [133]. This class of synthetic polymers is inexpensive and easy to prepare, allowing the scaling-up production of antimicrobial materials in the industry. Low molecular weight (MW) polymethacrylate derivatives polymers with relatively high yields were prepared avoiding the necessity of time-intensive fractionation of polymers by column chromatography [133]. A series of polymers of three different MW ranges displaying a wide range of mole percentages of butyl methacrylate (MPBu) (0%–60%) were tested for antimicrobial activity. The MICs for all series decreased as MPBu increased up to 30%, at which point further increase of the butyl functionality did not affect the MIC [135]. The smallest polymer series with 1,300–1,900 g·MW, further demonstrated the lowest MIC of 16 μg/mL above 30% MPBu. The increased activity with increasing MPBu percentiles suggested that the hydrophobicity of polymers is a major consideration in their antimicrobial action. As the polymers become more hydrophobic, incorporation of polymers to lipid membranes is enhanced, and thus the integrity of membrane is more efficiently disrupted [135]. However, similarly to antimicrobial peptides [136], further increases in MPBu percentiles create hydrophobic polymers more likely to undergo irreversible aggregation in water, preventing antimicrobial action [135].

Several cationic polymers are designed to mimic naturally occurring host-defense antimicrobial peptides which act on bacterial cell walls or membranes [137]. Antimicrobial-peptide mimetic polymers display antibacterial activity against a broad spectrum of bacteria including drug-resistant strains and are less susceptible to resistance development in bacteria. These polymers also show selective activity to bacteria over mammalian cells. Whereas peptide-mimetic antimicrobial polymers have the amino moiety, a low molecular weight and short alkyl chains, the polymer antimicrobial disinfectants have quaternary ammonium moieties, high molecular weight and long alkyl chains [137]. Table 3 illustrates these structural differences.

Recently, the role of the cationic moiety of the polymer on membrane disruption was evaluated [143]. Cationic polymers with primary amine groups induce reorganization of the bilayer structure to align lipid headgroups perpendicular to the membrane. The polymers bearing primary amines exceed the tertiary and quaternary ammonium counterparts in membrane binding and disrupting abilities due to complexation of primary amines to the phosphate groups in the lipids, through a combination of hydrogen bonding and electrostatic interactions [143].

Polyethylenimines (PEIs) range among the most potent transfection agents and hence constitute an interesting alternative to viral vectors for gene therapy [144]. Like other cationic polymers, PEIs display considerable antimicrobial activity. Synergistic antibacterial effects of PEI and antibiotics have been shown [145], and PEI has proven to be a valuable conjugate for photodynamic therapy of localized infections with Gram-positive and -negative bacterial, yeast, and fungal pathogens [146,147]. Furthermore, derivatives of PEI, which are effective in rupturing bacterial cell membranes, have also been suggested for antimicrobial coating of devices and surfaces [148]. Recently, a 25 kDa linear PEI was reported as a strong inhibitor of human papillomavirus and cytomegalovirus at concentrations much inferior to those toxic for mammalian cells in culture [149]. However, further studies are warranted to test optimized PEI derivatives for their microbicide potential and to investigate these compounds for antiviral efficiency in animal models.

PEI has also been used for the targeted antimicrobial photochemotherapy approach [150,151]. The conjugation between PEI and some photosensitizers such as porphirins or methylene blue derivatives produced interesting cationic molecules sensitive to light [150,151]. The targeted PDT approach against localized infections requires a nontoxic dye termed a photosensitizer (PS) and low-intensity visible light, which, in the presence of oxygen, produces cytotoxic species [150]. The photodynamic inactivation requires microbial exposure to visible light that causes the excitation of exogenous or endogenous PS molecules with the production of singlet oxygen and other reactive oxygen species able to react with intracellular components, and consequently produce cell inactivation. In contrast to polylysine conjugates, the PEI conjugates were resistant to degradation by proteases such as trypsin that hydrolyze lysine-lysine peptide bonds [147]. The advantage of protease stability combined with the ready availability of PEI suggests these molecules may be superior to polylysine-photosensitizers conjugates for photodynamic therapy (PDT) of localized infections [147].

Another class of interesting polymers sensitive to light that display antimicrobial properties are the poly(phenylene ethynylene) (PPE)-based cationic conjugated polyelectrolytes (CPEs) [152–158]. These PPE-based CPE including oligomeric (OPE) compounds are efficient broad-spectrum bactericides against both Gram-positive and Gram-negative bacteria. CPEs and OPEs exhibit remarkable light-activated biocidal activities. The light-induced biocidal activity of these compounds has also been attributed to the ability of their excited states to generate reactive oxygen species after activation with UV-visible light, which can strongly damage biomacromolecules. In the dark, however they also exhibit antimicrobial activity [159]. The molecular size of the antimicrobial compounds is one of the determining factors in their interactions with bacteria [160]. The relatively large sizes of the polymeric CPEs hinder their ability to penetrate into the cell wall and membrane, and as a result, they may only cause damages to the cell surfaces and cause cell aggregation. On the other hand, the smaller and unique linear structures of the oligomeric CPEs compounds enable them to easily penetrate cell walls and membranes without at first causing serious morphological changes to the cell surface. These oligomers may then exert their cytotoxicity by inducing small membrane defects and inhibiting metabolic pathways. The addition of the linear oligomers significantly decreases the optical density of *E. coli* cell suspensions [160]. Disintegration of bacterial cells is likely caused by the insertion of the linear oligomers into the cell walls and membranes and subsequent disruption of these structures. The more linear of the two tested oligomers showed the highest cell lysis activity, resulting in a large amount of cell debris that was both detected by absorbance measurements and visualized by scanning electron microscopy (SEM) [159].

Antimicrobial peptides (AMPs) are natural, amphiphilic sequences of 5–50 amino acid residues with net positive charges [161,162]. They are produced by bacteria, plants, and animals—both vertebrates and invertebrates [162–165]. Although AMPs produced by animals and plants and those produced by bacteria certainly function in entirely different settings, both represent a defensive approach against invading bacteria that compete with the AMP-producer for nutrients. These compounds are also considered to play an important role in innate immunity against microorganisms [166,167]. Whereas AMPs produced by bacteria are active at pico-to nanomolar concentrations, those produced by eukaryotes are active at micromolar concentrations [168]. They are small molecules with well defined hydrophobic and hydrophilic regions [162,169,170]. Upon contact with membranes they form separate patches rich in positively charged and hydrophobic amino acids. Folded peptides fall into four broad structural groups: β-sheet peptides stabilized by two to four disulfide bridges (for example, human α- and β-defensins, plectasin or protegrins); α-helical peptides (for example, LL-37, cecropins or magainins); extended structures rich in glycine, proline, tryptophan, arginine and/or histidine (for example, indolicidin); and loop peptides with one or disulfide bridge (for example, bacteriocins) [171]. Among the bacteriocins of Gram-positive bacteria, there is a particular group, the lantibiotics (lanthionine-containing peptide antibiotics), which are characterized by thioether-based intramolecular rings resulting from post-translational modifications of serine (or threonine) and cysteine residues (for example, nisin and mersacidin) [172]. Lanthionine rings, some of which represent conserved binding motifs for recognition of specific targets, create segments of defined spatial structures in the peptides which provide stability against proteases and against the antigen-processing machinery, since antibodies against highly cross-bridged antibiotics are very difficult to obtain [173].

AMPs can be nonribosomally (gramicidins, polymyxins, bacitracins, glycopeptides, *etc.*) or ribosomally synthesized. The former are often drastically modified and are largely produced by bacteria, whereas the latter are produced by all living species (including bacteria) as a major component of the natural host defense molecules of these species [174–176]. Ribosomally synthesized peptides are produced by eukaryotes and represent crucial components of their defense systems against microorganisms, being widely distributed in nature and produced by mammals, birds, amphibians, insects, plants, and microorganisms. Although they form a diverse group of peptides as judged by their primary structures, they are often cationic, amphiphilic and most of them kill bacteria by permeabilizing their cell membranes. Their positive charge presumably facilitates interactions with the negatively charged bacterial phospholipid membranes and/or acidic bacterial cell walls, whereas their amphiphilic character enables membrane permeabilization. Unfortunately, bacteria have found ways to modulate their anionic charges on the cell wall polymers or on the acidic phospholipids by introducing positively charged groups in their peptidoglycans, lipopolysaccharides, teichoic acids or membrane lipids. Two of these mechanisms involving the transfer of d-alanine into teichoic acids and of l-lysine into phospholipids, respectively, have been identified and characterized in *S. aureus*, a major human pathogen in community- and hospital-acquired infections. Inactivation of the responsible genes, dltABCD for alanylation of teichoic acids and mprF for lysinylation of phosphatidylglycerol, renders *S. aureus* highly susceptible to many human antimicrobial molecules and leads to profoundly attenuated virulence in several animal models [177]. Microbial resistance mechanisms against the host defense peptides include several strategies such as inactivation and cleavage of host defense peptides by production of host defense binding proteins and proteases, repulsion of the peptides by alteration of pathogen’s surface charge employing modifications by amino acids or amino sugars of anionic molecules (e.g., teichoic acids, lipid A and phospholipids), alteration of bacterial membrane fluidity, and elimination of the peptides using multi drug efflux pumps. Together with bacterial regulatory network(s) that regulate expression and activity of these mechanisms, they represent attractive targets for development of novel antibacterials [178–180].

While conventional antibiotics are active only against bacteria and/or fungi, AMPs have a broader range of applications against bacteria, fungi, parasites, enveloped viruses and cancer. A large variety of AMPs synthesized by bacteria belong to the group of bacteriocins which are not ribosomally synthesized [181]. Bacteriocins are small, heat stable peptides that bacteria use to compete against other bacteria of the same species (narrow spectrum) or against bacteria of other genera (broad spectrum) [182]. The majority of bacteriocins are active in the nanomolar range against Gram-positive bacteria in closely related species or in a broad-spectrum manner for many species. The most promising bacteriocins as antibiotics are produced by lactic acid bacteria (LAB) with the core genera including *Lactobacillus*, *Lactococcus*, *Leuconostoc*, *Pediococcus* and *Streptococcus*. Examples of such peptides are nisin [182,183], mersacidin, which is in pre-clinical test to treat Gram-positive infections [171] and lacticin (against mastitis infections) [184].

An essential feature for the cationic AMPs activity is the spatial separation of opposing hydrophobic and cationic domains, which allows their effective insertion into the negatively charged bacterial membrane. The synthesis of semi-synthetic analogues as small as three residues in length produced effective antimicrobial compounds, but their toxicity levels were high and there was a tight binding to hydrophobic domains of proteins such as serum albumin [185,186]. Modifying their hydrophobic domain affected these nonspecific interactions. For example, linking lipid tails to short peptide sequences was a convenient approach to rapid analogue synthesis [187]. However, the inherent protease susceptibility of the AMPs is an important factor that limits their use in therapeutics [186]. Peptoid residues are structurally similar to amino acids residues, but have the R-group transferred from the α-carbon to the amide nitrogen. The peptoids lack the ability to form backbone hydrogen bonds and standard peptide secondary structures but remain able to mimic AMPs activity [188]. Modified amino acid residues like peptoids are more resistant to cleavage by proteases, and have been used in the construction of a number of AMPS derivatives [188,189]. The lipopeptoids were found to have antimicrobial activity similar to that of previously reported lipopeptides, with the potential to avoid proteolysis by both human serum proteins and endogenously expressed bacterial proteases [190,191]. However, their toxicity towards eukaryotic cells was found to correlate to antimicrobial activity, with the most active antimicrobials significantly hemolytic as well [190].

## 3. The Antimicrobial Supramolecular Assemblies

Excellent review articles on antimicrobial polymers are available [192–196]. Their design and applications is an exponentially growing field, but their combination with appropriate inert or non inert vehicles to yield truly biocompatible, antimicrobial and supramolecular assemblies of low toxicity is still in its infancy. Biomimetic, biocompatible and hybrid antimicrobial supramolecular devices are urgently required to deliver bioactive molecules for a very diverse range of applications such as wound dressings, artificial organs, stem cells scaffoldings, food packing and preservation, tissue engineering, biofouling prevention, regenerative medicine and dentistry [197–204]. A major research effort in both academic laboratories and life science companies is being made to develop alternative antimicrobial strategies and products to which it is hypothesized bacteria will be unable to develop resistance. A truly antimicrobial biomimetics [42] is needed with films, coatings and surfaces, nanoparticles, liposomes, bilayers and novel hybrid materials specifically tailored for different applications in a variety of formulations. Various types of protein-based (wheat gluten, collagen, corn zein, soy, casein, and whey protein), polysaccharide-based (cellulose, chitosan, alginate, starch, pectin, and dextrin), and lipid-based (waxes, acylglycerols, and fatty acids) edible films and a wide range of antimicrobial agents have been used to enhance the safety and shelf life of foods [205,206]. Among these, chitosan has been recognized as a versatile cationic and antimicrobial biopolymer for producing biodegradable films and coatings, immobilizing enzymes and inducing hypocholesterolemic effect as a dietary supplement [207]. The development of effective assemblies including antimicrobial enzymes is also important to deal with the problems associated with biofilm formation in manufacturing, environmental protection and healthcare settings [208–210]. The enzymatic strategy allows degradation of microbial DNA, polysaccharides, proteins and quorum-sensing molecules [210]. For example, the protection of stainless steel surfaces against protein and/or bacterial adhesion was achieved from grafting of lysozyme and/or poly(ethylene glycol) onto the flat substrates pretreated with poly(ethylene imine) (PEI) [211]. The layer-by-layer (LbL) approach allowed the controlled release of an antimicrobial peptide, ponericin G1, from polyelectrolyte multilayer films which could be degraded by hydrolases and displayed excellent compatibility with NIH 3T3 fibroblasts and human umbilical vein endothelial cells [212]. In dispersion, the outermost layer of the cationic polymer PDDA displayed excellent microbicidal and fungicidal action at doses where haemolysis was absent [45,213]. Antimicrobial and haemolytic activities for PDDA alone or in assemblies of DODAB BF/CMC/PDDA are presented in Table 4.

**Figure 4:**
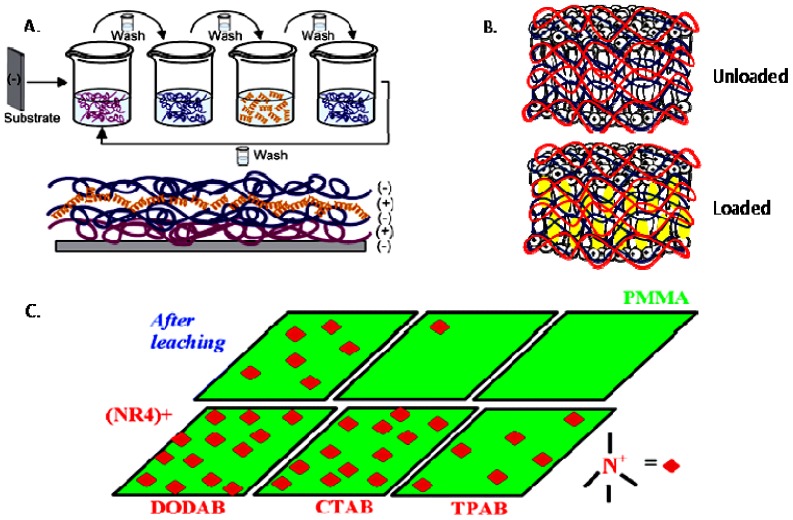
Self-assembled antimicrobial films and dispersions driven by the electrostatic or by the ion-dipole interaction. (**A**) The layer-by-layer (LbL) approach to optimize delivery of antimicrobial cationic peptide from films (adapted with permission from [212]); (**B**) Microbicidal PDDA polymer from microbicidal dispersions of cationic lipid/carboxymethylcellulose/polydiallyldimethylammonium bromide unloaded (adapted with permission from [45]) or loaded with yellow amphotericin B (adapted from [223]); (**C**) QACs impregnating PMMA films obtained by spin-coating also displayed bactericidal activity (adapted with permission from [216]).

Non-covalent association between polymers and antimicrobial quaternary ammonium compounds was achieved by several methods such as spin-coating or the LbL approach [214–216]. Intermolecular associations such as those driven by the electrostatic interaction in the LbL approach [217,218] or even the ion-dipole interactions between bioactive compounds and polymers [214] has been a major strategy to optimize the antimicrobial performance. Hybrid materials from the intermolecular associations between the antimicrobial cationic lipid DODAB and polymers such as polystyrene (PS) and poly(methyl methacrylate) (PMMA) were obtained from spin-coating of chloroformic solutions and characterized by ellipsometry, wettability, optical and atomic force microscopy, Fourier transform infrared spectroscopy (FTIR), differential scanning calorimetry (DSC), and activity against *E. coli* [214]. Whereas intermolecular ion-dipole interactions were available for the PMMA-DODAB interacting pair producing smooth PMMA-DODAB films, the absence of such interactions for PS-DODAB films caused lipid segregation, poor film stability (detachment from the silicon wafer) and large rugosity. In addition, the antimicrobial DODAB properties were transferred to the novel hybrid PMMA/DODAB coating, which was highly effective against *E. coli* [214]. Impregnation of polymers with quaternary ammonium compounds (QAC) was also achieved from deposition of alternate anionic and cationic polyelectrolyte layers where cetyltrimethylammonium bromide (CTAB) was the antimicrobial agent included in the cationic layer and film exposure to humidity allowed CTAB diffusion out of the film and bacterial growth inhibition in neighbouring regions [215]. Hybrid films from poly (methylmethacrylate) (PMMA) and dioctadecyldimethylammonium bromide (DODAB), cetyltrimethylammonium bromide (CTAB), or tetrapropylammonium bromide (TPAB) were characterized by determination of wettability, ellipsometry, atomic force microscopy, active compounds diffusion to water, X-ray photoelectron spectroscopy (XPS) with determination of atomic composition on the films surface, and biocidal activity against *Pseudomonas aeruginosa* or *S. aureus* [216]. QAC mobility in the films increased from DODAB to CTAB to TPAB. Diffusion and optimal hydrophobic-hydrophilic balance imparted the highest bioactivity to CTAB. DODAB sustained immobilization at the film surface killed bacteria upon contact. TPAB ability to diffuse was useless because of its unfavorable hydrophobic-hydrophilic balance for bioactivity as an antimicrobial agent [216]. Figure 4 illustrates the assembly of hybrid films or dispersions driven by the electrostatic or the ion-dipole interaction.

The one-pot, aqueous synthesis of cationic poly [2-(tert-butylaminoethyl) methacrylate] (PTBAM) nanofibers with embedded silver nanoparticles was used to reinforce the antimicrobial activity of silver [219]. The hybrid fibers displayed excellent antibacterial performance against *Escherichia coli* and *Staphylococcus aureus*. Furthermore, the prepared nanofibers exhibited enhanced bactericidal performance compared with the silver-embedded poly (methyl methacrylate) (PMMA) nanofibers, presumably because of the antibacterial properties of the PTBAM polymer [219].

The construction of anti-adhesive and antibacterial multilayer films from heparin and chitosan [220] was also improved by including silver ions which complexed with chitosan to yield a multifunctional assembly [221]. The modification of multilayered polymeric films with silver nanoparticles was recently reviewed in the literature [222].

The self-assembly of amphiphilic block copolymers can form a variety of nanostructures such as spherical or cylindrical micelles, vesicles and other more complex structures [224–227]. Polymer vesicles were obtained by simply adding the polymer powder into hot water, thanks to the partially hydrated nature of membrane-forming blocks [228,229]. Poly [2-(tert-butylaminoethyl) methacrylate] (PTA) is an effective cationic antimicrobial polymer with low toxicity against human cells [230,231]. Poly[2-(2-methoxyethoxy)ethyl methacrylate], PMeO2MA, with two ethylene oxide units as side groups, is soluble in water at room temperature and has good biocompatibility [232–234]. Recently, the diblock copolymer PMEO2MA-b-PTA was synthesized and the self-assembly of the copolymer molecules in water produced cationic polymeric vesicles [235]. These water soluble and antibacterial polymer vesicles without quaternary ammonium moieties or the loading of external antibiotics or nano-silvers have not been reported, which are more promising candidates in nanomedicine; the un-self-assembled polymer did not exhibit antibacterial activity [235].

The synergistic combination of pathogen surface recognition and lysis, in a engineered protein chimera led to protection against *Xylella fastidiosa* (Xf), a bacterium which causes diseases in multiple plants of economic importance [236]. One domain of this chimera is an elastase that recognizes and cleaves MopB, a conserved outer membrane protein of Xf. The second domain is a lytic peptide, cecropin B, which targets conserved lipid moieties and creates pores in the Xff outer membrane. A flexible linker joins the recognition and lysis domains, thereby ensuring correct folding of the individual domains and synergistic combination of their functions. The expression of the protein chimera in the xylem is able to directly target Xf, suppressing its growth, and decreasing the leaf scorching and xylem clogging in grapevines [236].

Cationic amphiphilic polymers were prepared based on a grafting-to strategy functionalizing branched polyethyleneimine (PEI) (MW: 25,000) by quaternary ammonium groups and alkyl chains of various chain length [237]. In a one-step addition, different functional ethylene carbonates were added to react with the primary amine groups of PEI. Ethylene carbonates substituted with different alkyl and quaternary ammonium groups were prepared. Both the surface activity and the membrane affinity were decreasing with increasing hydrophobicity of the molecules. The reason for that tendency was found to be not only the reduction of water solubility leading to aggregation with increasing hydrophobicity but also the unimolecular micelle formation which is observed at lower concentration well below the aggregation concentration. This conformational change expressed mainly in polymers coupled with alkyl chains of 14 and 16 carbon atoms made less accessible the hydrophobic chains for interaction with lipid layer. That effect results in reduction of the otherwise considerable membrane affinity [237].

**Figure 5:**
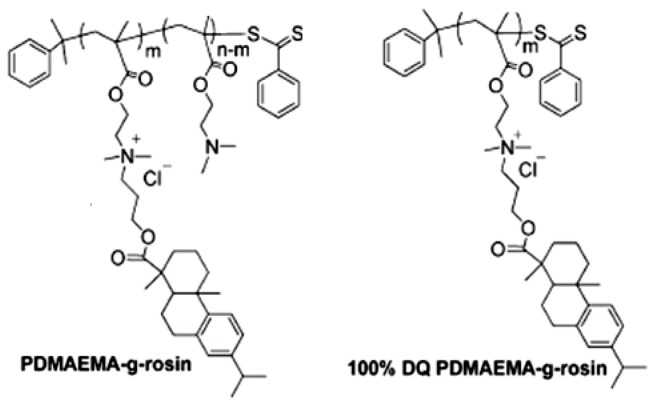
Chemical structure of rosin substituted polyesters with quaternary ammonium sandwiched between the polymer backbone and hydrophobic hydrophenanthrene [247].

Poly(*N*,*N*-dimethylaminoethyl methacrylate) (PDMAEMA) based-copolymers usually exhibit bactericidal activity and are often used to inhibit the growth of bacteria on different materials such as glass, filter paper, and plastics [238–241]. Quaternization of PDMAEMA or its copolymers, generally employing an alkylquaternized derivative, is often used to improve antibacterial activity by increasing positive charge density and amphipathic character of these polymers [242,243]. On the other hand, rosin, also referred to as resin acids or rosin acids, is a natural product derived from pine trees, and its derivatives have been widely used as additives and modifiers for various applications such as tackifiers, surfactants, antifouling coatings, food additives, *etc.* [244]. These natural products have a characteristic bulky hydrophenanthrene moiety. Rosin acids have received much attention as a renewable feedstock in polymer synthesis [244]. Recently rosin-substituted polyesters with quaternary ammonium (QA) group located at the periphery [245] or sandwiched between the polymer backbone and bulky hydrophobic hydrophenanthrene side groups were obtained [246]. These polymers showed excellent biocompatibility and antibacterial activity [245,246]. Their chemical structure is illustrated in Figure 5.

Ceragenins are cholic acid derivatives that mimick antimicrobial peptides. The ceragenin Cationic Steroidal Antimicrobial-13 or CSA-13 is a synthetic analog of a novel broad-spectrum AMP. This CSA-13 bactericidal molecule acts similarly to AMPs [247], however, ceragenins are less likely to be deactivated through associations with mucins, DNA, and F-actin as compared to the natural AMPs [247,248]. Furthermore, because CSA-13 is not peptide based and cannot be a substrate for proteases, it is stable under physiological conditions. CSA-13 mitigates antibiotic resistance by introducing a novel approach to eliminate bacteria that is not dependent upon molecular binding with receptor sites on the bacterial membrane. When critical concentrations of CSA-13 accumulate, the bacterial membrane is rapidly depolarized and cell death soon follows. It is believed that the cationic portions of the molecule insert themselves into the negatively charged bacterial cell wall causing a severe disruption that subsequently kills the microorganism [249].

In several types of infections, bacteria encounter unfavourable conditions that cause cells to enter into a quiescent state of slow growth or even no growth. These metabolically inactive organisms can survive high concentrations of antibiotics, and extended treatment is therefore required for drug efficacy. When the drug concentration falls below levels that kill or inhibit the growing cells, dormant bacteria can reactivate to become growing cells, and the host begins to show symptoms of disease again. A consensus is emerging that persisting bacteria evade antibiotic killing by substantially downregulating the biosynthetic processes that are targeted by most antibiotics without affecting bacterial survival in the metabolically inactive state [250–252]. For example, β-lactam antibiotics kill by activating autolysins and this requires active peptidoglycan synthesis in cells undergoing division. Agents as the cationic surfactants, lipids, polymers or CSA-13 that disorganize the structure of the membrane or that inhibit respiratory enzymes involved in energy production and establishing the membrane’s proton motive force are valid approaches for treating infections containing quiescent bacteria [252]. Biofilm-mediated infections and tuberculosis (TB) provide two major examples of unmet clinical need owing to bacteria that are either slow growing or dormant and therefore hard to treat [253]. Immunological tests provide evidence of latent tuberculosis in one third of the global population, which corresponds to more than two billion individuals. Latent tuberculosis is defined by the absence of clinical symptoms but carries a risk of subsequent progression to clinical disease, particularly in the context of co-infection with HIV [253].

Recently, the *in vitro* performance of CSA-13 embedded in silicone polymer for challenges against methicillin-resistant *Staphylococcus aureus* (MRSA) was investigated [254]. A weight-to-weight (*w*/*w*) concentration of 18% CSA-13 in silicone eliminated 5 × 10^8^ CFU of MRSA within 8 h [254]. A review on immobilization of antimicrobial agents both for medical and food preservation applications discussed the avoidance of toxicity allowed by immobilized biocides and the compatibility and reservoir limitations common to elutable agents [255].

**Figure 6:**
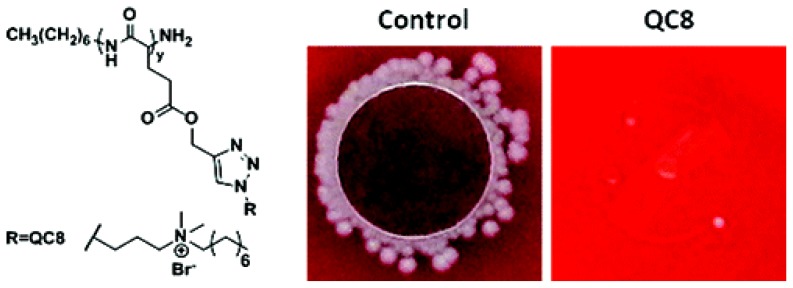
Antimicrobial peptide (AMP)-mimetic polypeptide QC8 bearing an alkyl quaternary ammonium moiety in its chemical structure. Adapted with permission from [256].

A library of antimicrobial polypeptides was easily prepared by the ring-opening polymerization of γ-propargyl-l-glutamate *N*-carboxyanhydride and the alkyne-azide cycloaddition click reaction, which mimic the favorable characteristics of naturally occurring antimicrobial peptides (AmPs) [256]. The polypeptides ranged in length from 30 to 140 repeat units with variable side group functionalities, including primary, secondary, tertiary, and quaternary amines with hydrocarbon side chains ranging from 1 to 12 carbons long and exhibited broad-spectrum activity against both Gram positive and Gram negative bacteria, namely, *S. aureus* and *E. coli*, while having very low hemolytic activity [256]. Figure 6 illustrates the chemical structure and antimicrobial activity of QC8, one of these quaternary ammonium derivatives AMPs such as QC8.

Numerous chemical structures have been designed and synthesized to mimic the cationic and amphiphilic structure of natural AMPs such as those of synthetic peptides [256–258], peptoids [188–191], polymethacrylates [259], polypyridines [260], polynorbornenes [261], polysaccharides [262], *etc.* Many synthesis efforts have considered the interaction of the antimicrobial peptides/polymers with the microbe cytoplasmic membrane but have largely ignored their compatibility with the complex cell wall structure and the peptidoglycan layer. Recently, peptidopolysaccharides, specifically a copolymer of chitosan and polylysine, were designed as peptidoglycan-mimetic compounds able to penetrate into the bacterial cell wall resulting in much reduced cytotoxicity to mammalian cells [263] in contrast with several hemolytic AMPs and AMPs analogues [259].

A series of cationic poly (sulfone amines) (PSAs) with different branched architectures and their polymer/silver (PSA/Ag) nanocomposites were synthesized through the polycondensation-addition reaction of divinylsulfone and 1-(2-aminoethyl)piperazine in mixed solvents [264]. The silver ions were complexed to PSAs and then reduced to yield PSA/Ag nanocomposites. The size of the silver nanoparticles (AgNPs) decreased with an increasing polymeric branched architecture [264]. The effect of the branched architecture on the antimicrobial activity was quite different for PSAs and PSA/Ag nanocomposites. For PSAs, the antimicrobial activity decreased with the branched architecture due to the reduced zeta-potential and low toxicity of the branched polymers. PSA/Ag nanocomposites exhibited an enhanced antimicrobial activity with an increasing polymeric branched architecture due to the high specific surface of small AgNPs [264]. The cytotoxicity of PSAs decreased with an increasingly branched architecture due to decreasing molecular volume and compact spatial structure, as well documented from the hydrodynamic volume of a polymer decreasing with an increasing branched density at similar molecular weights [265].

A series of poly(sodium styrene sulfonate)-b-poly(methyl methacrylate), PSSNa-b-PMMA, amphiphilic diblock copolymers have been synthesized through atom transfer radical polymerization (ATRP) of Me methacrylate (MMA) in *N,N*-dimethylformamide/water mixtures, starting from a PSSNa macroinitiator [266]. These diblock copolymers were shown to self-assemble in water forming micellar structures characterized by an increasing hydrophobic character and a decreasing size as the length of the PMMA block increases. These micelle-like structures turn from surface inactive to surface active as the length of the PMMA block increases. Moreover, contrary to the MMA-rich random copolymers, the respective diblock copolymers form water insoluble polymer/surfactant complexes with cationic surfactants such as hexadecyltrimethyl ammonium bromide (HTAB), leading to materials with antimicrobial activity [266]. This work agrees with the previously reported ability of PMMA polymer to combine with antimicrobial cationic surfactants and lipids via ion-dipole interaction producing antimicrobial materials [215,217].

Several inert materials have also been used in combination with antimicrobial polymers as much as inert polymers have been used in combination with antimicrobial materials. Recent examples are cationic polymer modified silica nanoparticles with enhanced antibacterial performance compared to bulk polycations and reduced adhesion on the surface of glass [267], non-leaching antimicrobial polyamide nanocomposites based on organoclays modified with a cationic polymer [268], cationic β-cyclodextrin-based polymers complexed with antibiotics such as butylparaben and triclosan and used to adsorb onto cellulose fibers; these affected the metabolism of the bacteria instead of damaging the cell membrane [269], antimicrobial and cationic polyhexamethylene guanidine hydrochloride (CPHGH) assembled to temperature-responsive, acetalyzed poly(vinyl alc.)/sodium acrylate (APVA-co-AANa) as multilayers [270], copolymers of 4-vinyl-*N*-hexylpyridinium bromide and dimethyl(2-methacryloyloxyethyl) phosphonate self-assembled onto titanium surfaces to form biocompatible and antimicrobial ultrathin layers able to prevent biofilm formation on implants [271], chitosan formulations, complexes and derivatives with other substances able to prevent or treat wound and burn infections not only because of its intrinsic antimicrobial properties, but also by virtue of its ability to deliver extrinsic antimicrobial agents to wounds and burns [200], hydrogels, formed by cationic polymers alone, or polymer mixtures with antimicrobial surfactants, lipids, nanoparticles, peptides, antibiotics or antiviral drugs [272], metallic-based micro and nano-structured materials such as copper, zinc and titanium and their oxides assembled into polymers with the migration of cations from the polymer matrixes determining their antimicrobial effectiveness [273–275], nonwoven poly(ethylene terephthalate) assembled with cationic quaternary ammonium antimicrobial polymer [276], rayon fibres made antimicrobial and thermal-responsive via layer-by-layer assembly with the appropriate functional polymers [277], silver nanoparticles capped with diaminopyridinylated heparin (DAPHP) and hyaluronan (HA) polysaccharides [278], *etc.*

While some natural antibacterial materials, such as zinc and silver, possess greater antibacterial properties as particle size is reduced into the nanometer regime (due to the increased surface to volume ratio of a given mass of particles), the physical structure of a nanoparticle itself and the way in which it interacts with and penetrates into bacteria appears to also provide unique bactericidal mechanisms [274]. Cationic nanoparticles were shown to induce the formation and/or growth of nanoscale holes in supported lipid bilayers. Noncytotoxic concentrations of cationic nanoparticles induce 30–2,000 pA currents in human embryonic kidney and human epidermoid carcinoma cells, consistent with a nanoscale defect such as a single hole or group of holes in the cell membrane [279,280]. The cell membrane leakage observed at non-cytotoxic concentrations arises from the nanoscale holes induced in the cell plasma membrane [279]. Bacteria and lipid vesicles were used to study the effect of a cationic sequence-random copolymer having an average length of 21 residues that is active against both Gram-positive and Gram-negative bacteria [281]. At low concentrations, this polymer is able to permeabilize model anionic membranes that mimic the lipid composition of *E. coli*, *S. aureus*, or *Bacillus subtilis* but is ineffective against model zwitterionic membranes, which explains its low hemolytic activity. The polymer is capable of binding to negatively charged vesicles, inducing segregation of anionic lipids. The appearance of anionic lipid-rich domains results in formation of phase-boundary defects through which leakage can occur. At low concentrations, the polymer permeabilizes the outer and inner membranes; at higher polymer concentrations, permeabilization of the outer membrane is progressively diminished, while the inner membrane remains unaffected with the polymer blocking the passage of solutes into the intermembrane space [281].

In general, positive charge on macromolecules (e.g., dendrimers) or nanoparticles appears to improve the efficacy of imaging, gene transfer, and drug delivery but the higher cytotoxicity of such constructs remains a major concern. Cationic nanoparticles (NPs) cause more pronounced disruption of plasma-membrane integrity, stronger mitochondrial and lysosomal damage, and a higher number of autophagosomes than anionic NPs [282]. In general, nonphagocytic cells ingest cationic NPs to a higher extent, but charge density and hydrophobicity are equally important; phagocytic cells preferentially take up anionic NPs. Cells do not use different uptake routes for cationic and anionic NPs, but high uptake rates are usually linked to greater biological effects [282]. The different uptake preferences of phagocytic and nonphagocytic cells for cationic and anionic NPs may influence the efficacy and selectivity of NPs for drug delivery and imaging. For antimicrobial chemotherapy using the antimicrobial polymers and their assemblies, a scrutinity of cytotoxic effects on different cell types and on *in vivo* animal models will be required before biomedical applications can be achieved. Furthermore, there are reports on development of resistance against cationic antimicrobial polymers [283]. The cationic antimicrobial polymer poly(2-(dimethylamino ethyl)methacrylate) (pDMAEMA) was more effective at antagonizing growth of clinical isolates of *Staphylococcus epidermidis* than of *S. aureus* and this was due to the fact that the *S. aureus* isolates tested were generally more hydrophobic than the *S. epidermidis* isolates and had a less negative charge [283]. A high content analysis of cytotoxic effects of pDMAEMA on human intestinal epithelial and monocyte cultures revealed several toxicological aspects of the cationic polymer [284]. Following 24–72 h exposure, 25–50 μg/mL pDMAEMA induced necrosis in U937 cells, while 100–250 μg/mL induced apoptosis in Caco-2. Increasing pDMAEMA concentration, decreased [Ca^2+^]i in U937 cells and increased [Ca^2+^]i in Caco-2 cells. Phospholipidosis was not observed in either cell line. pDMAEMA (10 mg/mL) did not induce any histological damage on rat colonic tissue and only mild damage to ileal tissue following exposure for 60 min the rathr complete toxicological analysis showed that pDMAEMA induces cytotoxicity in different ways on different cell types at different concentrations [284].

A biodegradable and *in vivo* applicable antimicrobial polymer was recently synthesized by metal-free organocatalytic ring-opening polymerization of functional cyclic carbonate in form of nanoparticles [285]. These nanoparticles disrupt microbial walls/membranes selectively and efficiently, thus inhibiting the growth of Gram-positive bacteria, methicillin-resistant *Staphylococcus aureus* (MRSA) and fungi, without inducing significant haemolysis over a wide range of concentrations [285]. These biodegradable nanoparticles, which can be synthesized in large quantities and at low cost, are promising as antimicrobial drugs, and can be used to treat MRSA-associated infections, which are often linked with high mortality.

**Figure 7:**
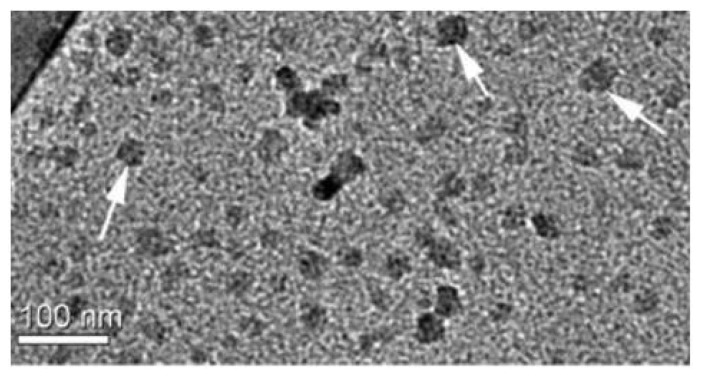
Cryo-transmission electron micrograph of bovine serum albumin (BSA)/PDDA coacervates at pH 8.5 and 100 mM of ionic strength. Adapted with permission from [287].

Other less explored approach regards the assembly of antimicrobial polymers with biocompatible and biodegradable vehicles such as proteins or polyssacharides to produce self-assembled nanoparticles [45,223,286–288]. There has been a great interest in application of nanoparticles as biomaterials for delivery of therapeutic molecules such as drugs and genes, and for tissue engineering. In particular, biopolymers are suitable materials as nanoparticles for clinical applications due to their versatile traits, including biocompatibility, biodegradability and low immunogenicity. Biopolymers are polymers that are produced from living organisms, generally classified in three groups: proteins, polyssacharides and nucleic acids. It is important to control particle size, charge, morphology of surface and release rate of loaded molecules to use biopolymer-based nanoparticles as delivery carriers for antimicrobial polymers. To obtain a nano-carrier for therapeutic purposes, biocompatible nanoparticles consisting of biopolymers such as protein (silk, collagen, gelatin, β-casein, zein and albumin) and protein-mimicked polypeptides and polysaccharides (chitosan, alginate, pullulan, starch and heparin) have been described and recently reviewed [286,289]. Electrostatic interactions between synthetic antimicrobial polymers and proteins can lead to the formation of dense, macroion-rich liquid phases, with equilibrium microheterogeneities on length scales up to hundreds of nanometers [287]. For example, coacervates obtained for bovine serum albumin (BSA) and chitosan or for BSA and poly (diallyldimethylammonium chloride) (PDDA), a cationic and synthetic antimicrobial polymer [45], are essentially nanoparticles as illustrated in Figure 7.

In Table 5, there are some examples of cationic polymers and polypeptides, and their antimicrobial and haemolytic toxicity.

As the polymers become more hydrophobic, their incorporation to the lipid membrane is enhanced, and thus the integrity of bacterial membrane is more efficiently disrupted [135]. In Table 5, the highest hydrophobicity and the highest antimicrobial efficiency against Gram-negative, -positive or fungus can be ascribed to PDDA. One should notice that only for PDDA the MBC values were available in the literature [45,213]. For the other polymers, only MIC values were retrieved. Furthermore, PDDA is a well-known polymer. PDDA has been considered safe for human health and is widely used in paper manufacturing, water treatment, mining industries, besides biological or medical applications and food processing [290]. The cell envelopes of most bacteria fall into one of two major groups. Gram-negative bacteria are surrounded by a thin peptidoglycan cell wall, which itself is surrounded by an outer membrane containing lipopolysaccharide. Gram-positive bacteria lack an outer membrane but are surrounded by layers of peptidoglycan many times thicker than is found in the gram-negatives. Threading through these layers of peptidoglycan are long anionic polymers, called teichoic acids. The teichoic acids are responsible for the anionic properties of the bacterial cell surface [179]. In resistant strains, the teichoic acid may be replaced by alanine residues conferring reduced negative charge to the cell wall [179]. On the other hand, the lipopolyssacharides present in the complex structures of Gram-negative bacteria confer negative charges to the surface [291]. Among the polymers in Table 5, penetration in the outer membrane of the Gram-negative bacteria was optimal for PDDA due to its high hydrophobicity, whereas the other cationic polymers, more hydrophilic than PDDA, required much higher doses to exhibit activity. Against mammalian cells, such as red blood cells, most cationic polymers displayed very high percentile of haemolysis in comparison to PDDA (Table 5). Important features of polymers useful as disinfectants are not only their microbicidal potency but also low toxicity to human cells, especially for medical/clinical use. Considering this, and the characteristics of the cationic polymers listed above, the ideal polymers and their assemblies are those which display excellent activity against microorganisms and practically no toxicity to red blood cells. PDDA fits in these characteristics, as much as its hybrid assemblies of DODAB BF/CMC/PDDA, already described in Table 4 [45,213].

## 4. Conclusions

Macromolecular antimicrobial agents such as cationic polymers, peptoids, peptides and lipopeptides have been under an increased level of scrutiny because they can combat multi-drug-resistant microbes. However, most of these polymers are neither biocompatible nor biodegradable. The straightforward ways of avoiding toxicity would be the design of biodegradable novel materials or the use of natural cationic polymers (e.g., chitosan). Other approaches to be further explored involve the combinations of antimicrobial polymers and appropriate materials either by grafting or by self-assembly in order to produce formulations with adequate performance and low toxicity *in vivo*. Biopolymers such as polysaccharides and proteins or even biocompatible synthetic polymers such as PMMA could be good alternatives for combinations with antimicrobial polymers aiming at antimicrobial chemotherapy *in vivo*. The use of antimicrobial macromolecular agents, however, is not restricted to *in vivo* applications: they may also be used *ex vivo* for water disinfection, food packing and preservation and many antifouling applications.

## Figures and Tables

**Table 1:** Examples of cationic compounds and assemblies displaying antimicrobial properties.

Cationic molecule or assembly	Name	References
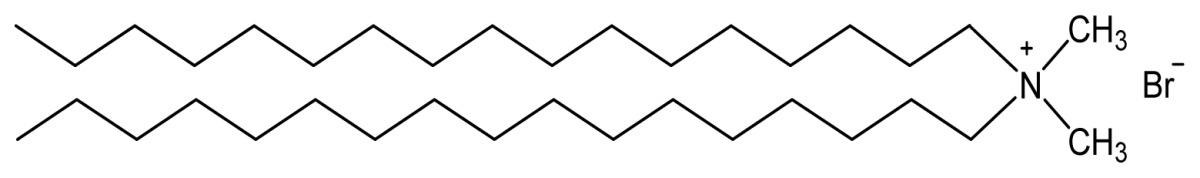	Dioctadecyldimethylammonium bromide (DODAB)	[16,17,23–27]
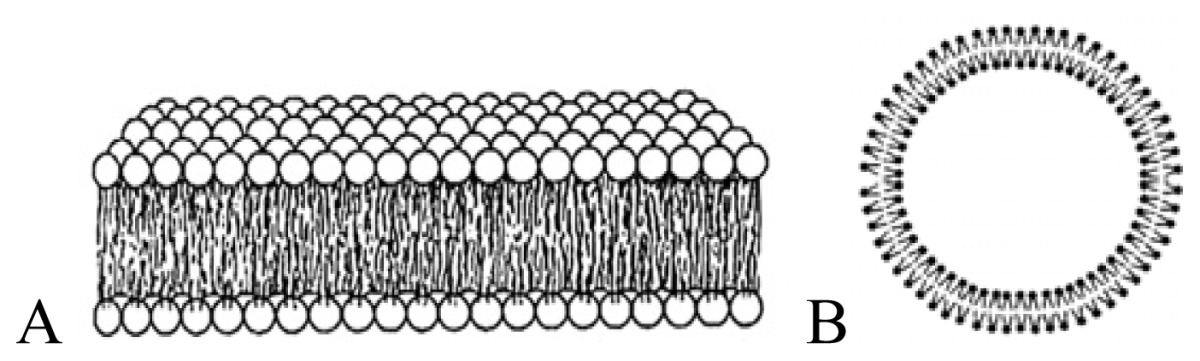	Cationic bilayer fragment (A) Large cationic vesicle (B)	[36–42]
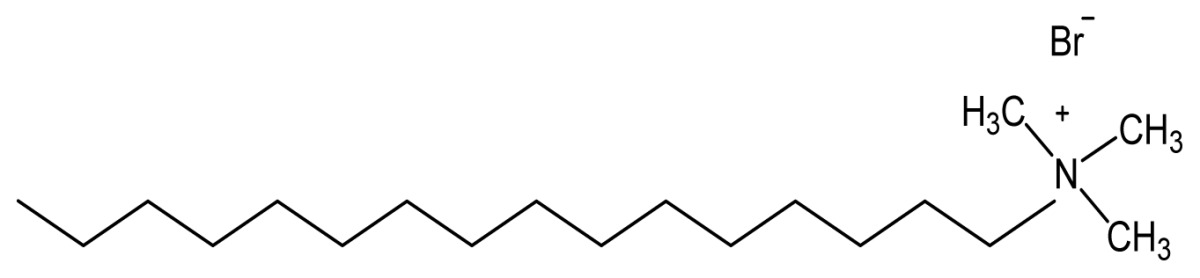	Hexadecyltrimethylammonium bromide (CTAB)	[43,44]
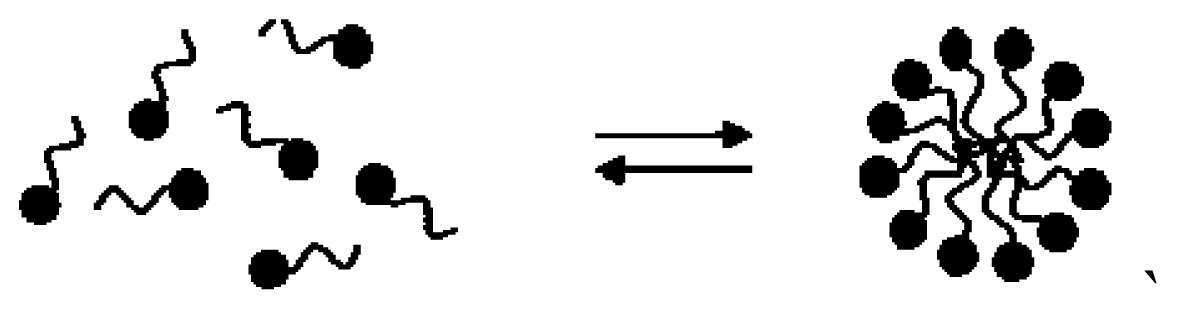	CTAB micelle	[43,44]
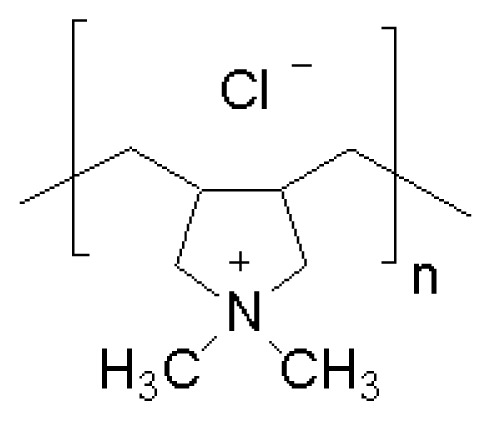	Poly (diallyldimethyl) ammonium chloride (PDDA)	[45]
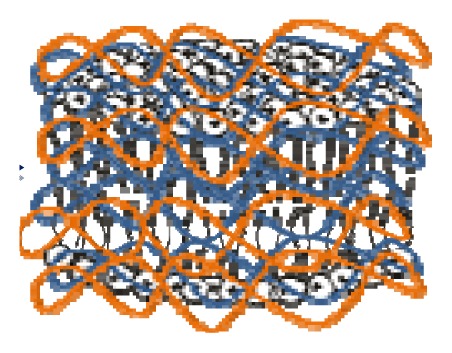	Cationic bilayer fragment/carboxymethylcellulose (CMC)/PDDA nanoparticle	[45]
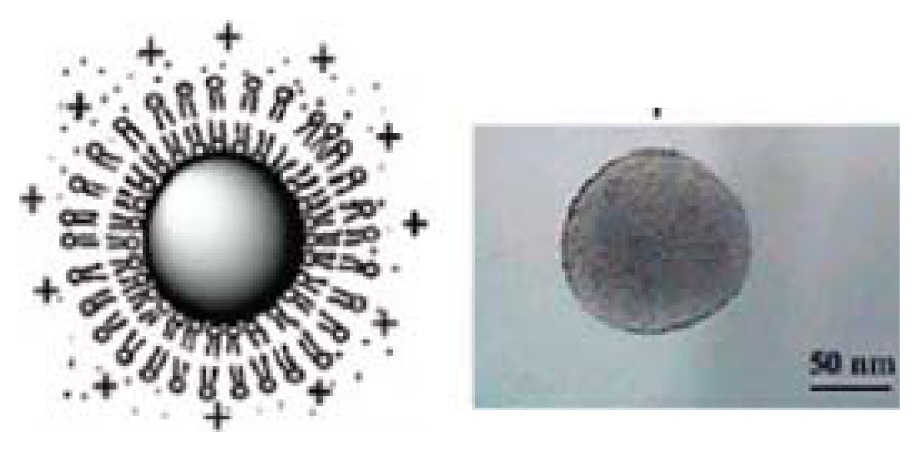	Polystyrene sulfate nanoparticle supporting a DODAB cationic bilayer	[46,47]

**Table 2:** Differential cytotoxicity of dioctadecyldimethylammonium bromide (DODAB) cationic lipid assembled as bilayer fragments. Interaction time between DODAB bilayer fragments and cells was 1 h or * 48 h.

Cell type	Cells/mL	[DODAB], mM (μg/mL)		References

		50% survival	0% survival	
**Mammalian cells**				
Kidney epithelial cells	10^5^	5.4 (3,400)	-	[27]
3T3(cloneA31) fibroblasts	10^4^	1.0 (631)	-	[109,110]
SV40-SVT2 fibroblasts	10^4^	1.0 (631)	-	[109,110]
**Gram-negative bacteria**				
*E. coli*	2 × 10^7^	0.028 (17.7)	-	[16,17,23,24,109]
*S. typhimurium*	2 × 10^7^	0.010 (6.3)	-	[16,17,109]
*P. aeruginosa*	3 × 10^7^	0.005 (3.2)	-	[16,17,109]
**Gram-positive bacteria**				
*S. aureus*	3 × 10^7^	0.006 (3.8)	-	[16,17,109]
**Yeasts**				
*C. albicans* ATCC 90028 *	10^3^	-	0.4 (250)	[27]
*C. albicans* ATCC 90028	2 × 10^6^	0.010 (6.3)	-	[25,109]

**Table 3:** Examples of synthetic peptide, peptoide, polymer, copolymer and peptide-mimetic polymer displaying antimicrobial properties.

**Synthetic peptide ref. [138]**	**Antimicrobial polymer ref. [45]**
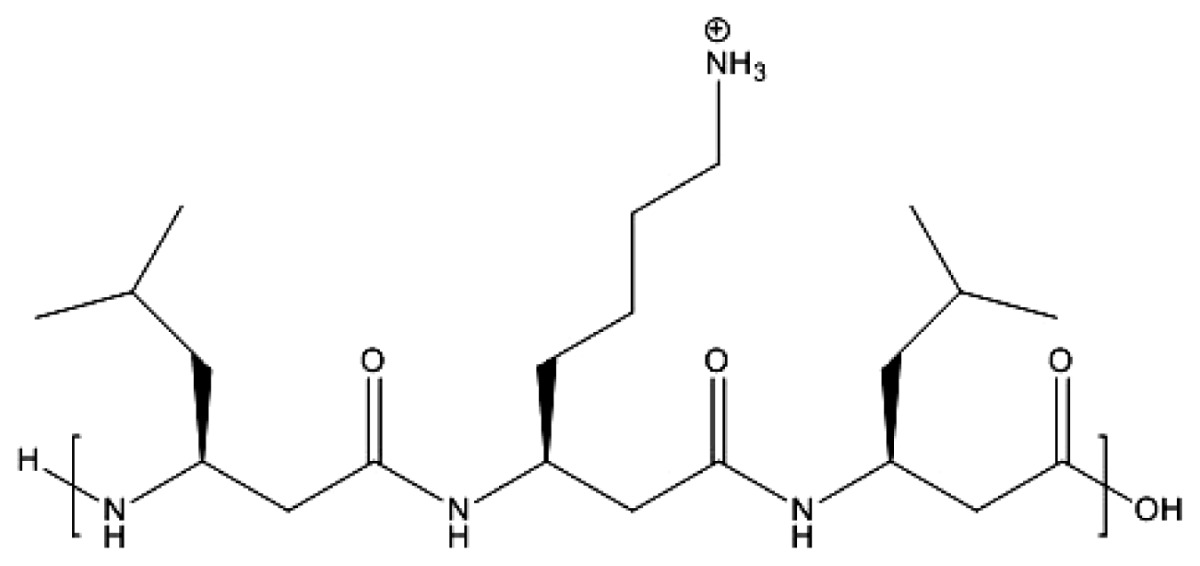	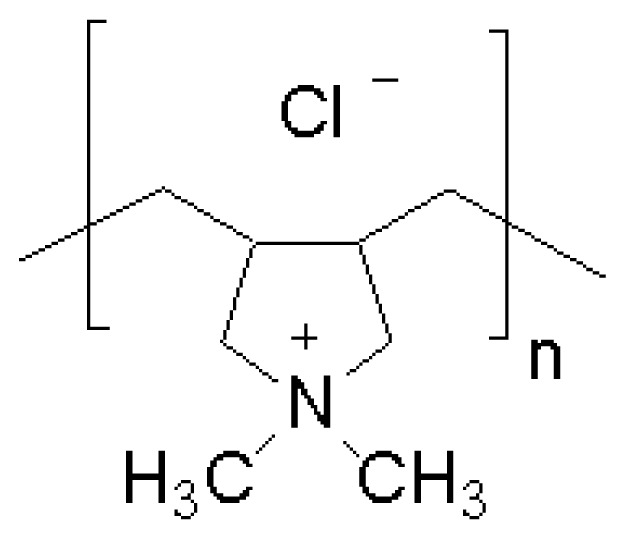

**Synthetic peptoid ref. [139]**	**Antimicrobial copolymer ref. [140]**
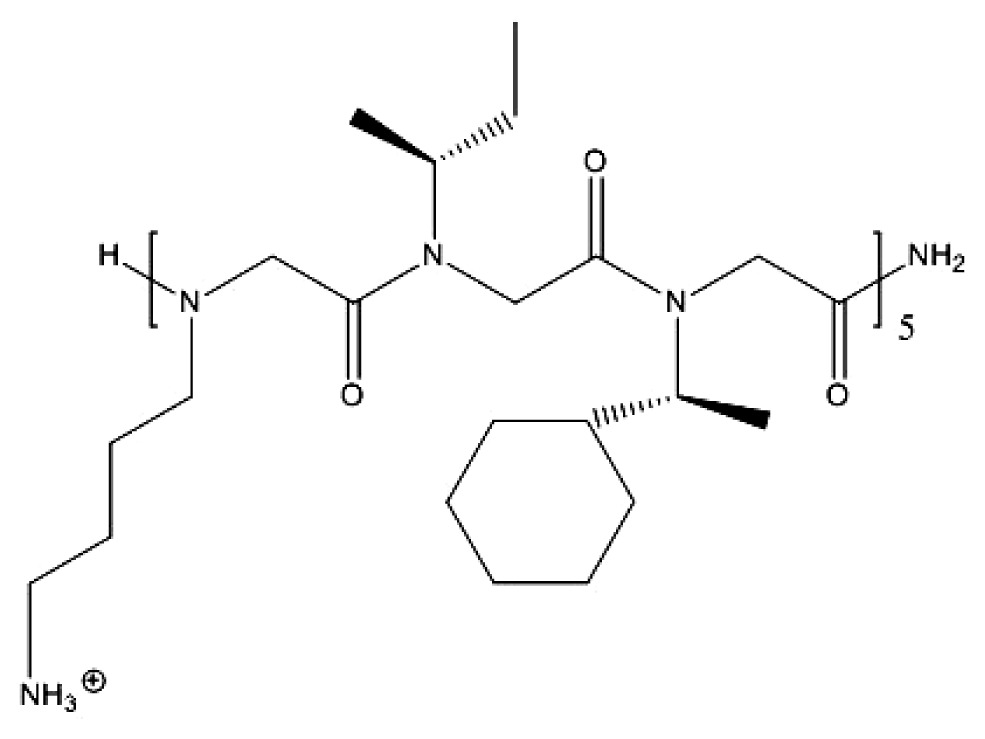	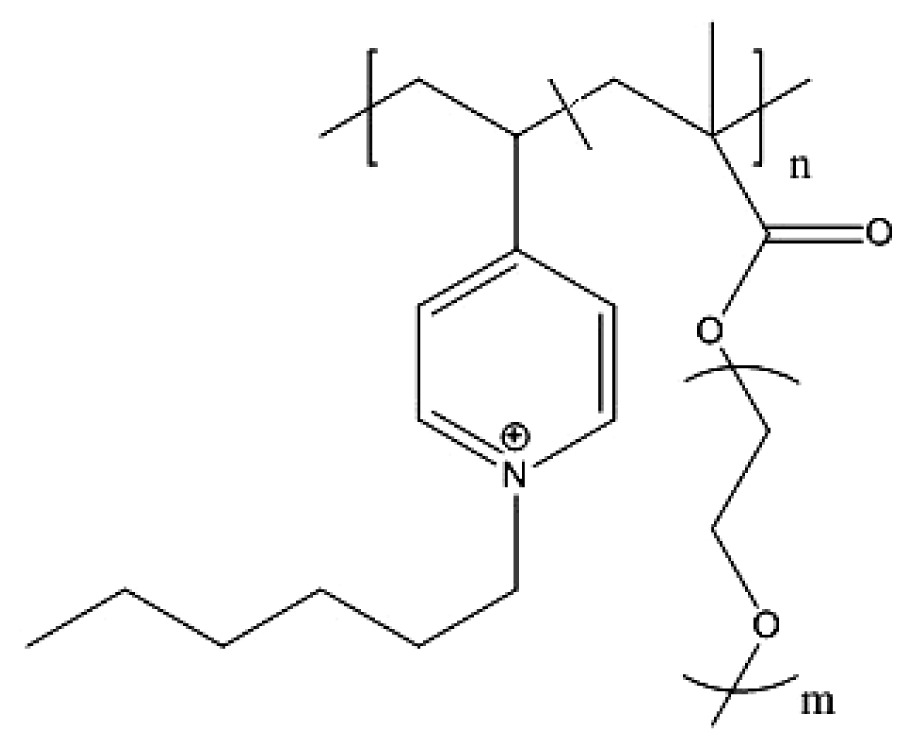

**Peptide-mimetic antimicrobial polymers ref. [91,141,142]**	
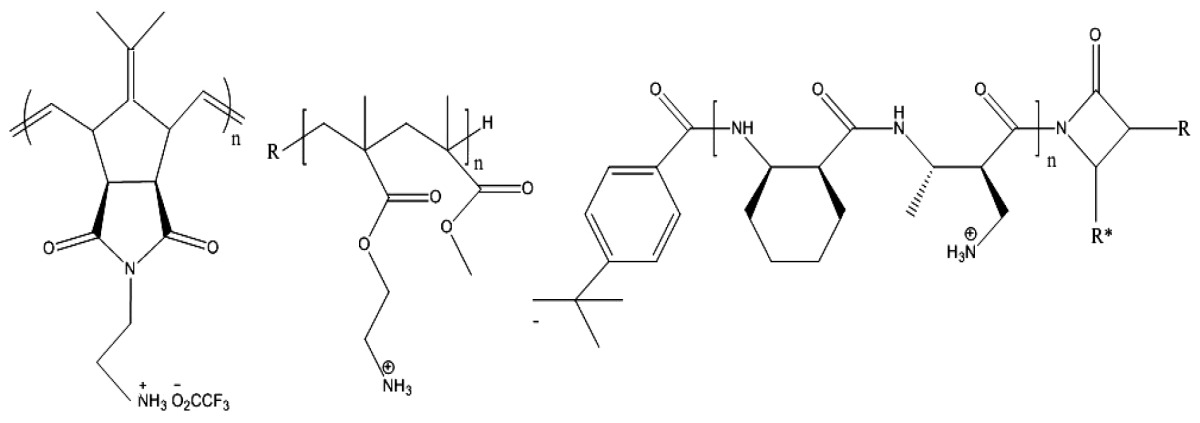	

**Table 4:** Minimum Bactericidal or Fungicidal Concentrations (MBC or MFC) and % haemolysis for hybrid assemblies of DODAB BF/CMC/PDDA (Small and Large Particles, SP and LP). Haemolysis was determined for 1 h of interaction time at 100 and 63 μg/mL poly (diallyldimethylammonium chloride) (PDDA) and DODAB, respectively.

Dispersion	MBC or MFC (μg/mL)						% Haemolysis ref. [213]

	[PDDA]	[DODAB]	[PDDA]	[DODAB]	[PDDA]	[DODAB]	

	*P. aeruginosa* ref. [45]		*S. aureus* ref. [45]		*C. albicans* ref. [213]		
SP	2.0	1.0	10.0	6.0	0.5	0.32	0
LP	2.0	1.0	10.0	6.0	1.0	0.63	0

**Table 5:** Minimum Inhibitory Concentrations (MIC) and haemolysis for some cationic polymers.

Polymer	Microrganism	MIC (μg/mL)	Haemolysis	Ref.
Polyionene 5	*P. aeruginosa*	100–330	Nt	[86]
	*S. aureus*	5–10		

Polyionene 14	*P. aeruginosa*	330–660	Nt	[86]
	*S. aureus*	33–66		

Lipopeptoid	*P. aeruginosa*	64	56% (at 100 μg/mL lipopeptoid)	[190]
C16N_lys_GN_lys_	*S. aureus*	8		

Lipopeptoid	*P. aeruginosa*	128	68% (at 100 μg/mL lipopeptoid)	[190]
C16N_lys_N_lys_N_lys_	*S. aureus*	16		

Lipopeptide	*P. aeruginosa*	* 128	4% (at 100 μg/mL lipopeptide)	[191]
C16-KKK	*S. aureus*	* 16		

Lipopeptide	*P. aeruginosa*	* 64	63% (at 100 μg/mL lipopeptide)	[191]
C16-KGK	*S. aureus*	* 8		

pDMAEMA	*P. aeruginosa*	1000	50%	[242]
	*S. epidermidis*	100	>10 mg/mL	

PDDA	*P. aeruginosa*	** 1.0	0% (at 100 μg/mL)	[45,213]
	*S. aureus*	** 10		
	*C. albicans*	** 0.5		

* MIC for 90% inhibition of microorganisms.

** MBC for 99% of cell death. Nt: Not tested.

## References

[1] McDonnell G., Russell A.D. (1999). Antiseptics and disinfectants: Activity, action, and resistance. Clin. Microbiol. Rev.

[2] Larson E.L., Olmstad R.N. (1996). Antiseptics. APIC Infection Control & Applied Epidemiology: Principles & Practices.

[3] Rutala W.A., Weber D.J. (2004). Disinfection and sterilization in health care facilities: What clinicians need to know. Clin. Infect. Dis.

[4] Block S.S., Block S.S. (2001). Historical Review. Disinfection, Sterilization, and Preservation.

[5] Frier M., Hugo W.B. (1971). Derivatives of 4-Amino-quinaldinium and 8-Hydroxyquinolone. Inhibition and Destruction of the Microbial Cell.

[6] Merianos J.J., Block S.S. (1991). Quaternary Ammonium Antimicrobial Compounds. Disinfection, Sterilization, and Preservation.

[7] Hugo W.B., Frier M. (1969). Mode of action of antibacterial compound dequalinium acetate. Appl. Microbiol.

[8] Hugo W.B., Russell A.D., Hugo W.B., Ayliffe G.A.J. (1999). Disinfection Mechanisms. Principles and Practice of Disinfection, Preservation and Sterilization.

[9] Salton M.R.J. (1968). Lytic agents, cell permeability and monolayer penetrability. J. Gen. Physiol.

[10] Denyer S.P. (1995). Mechanisms of action of antibacterial biocides. Int. Biodeterior. Biodegradation.

[11] Davies A., Field B.S. (1969). Action of biguanides, phenols and detergents on *Escherichia coli* and its sphereoplasts. J. Appl. Bacteriol.

[12] Kanazawa A., Ikeda T., Endo T. (1995). A novel approach to mode of action of cationic biocides: Morphological effect on antibacterial activity. J. Appl. Bacteriol.

[13] Russell A.D., Chopra I. (1996). Understanding Antibacterial Action and Resistance.

[14] Cabral J.P.S. (1992). Mode of antibacterial action of dodine (dodecylguanidine monoacetate) in *Pseudomonas syringae*. Can. J. Microbiol.

[15] Codling C.E., Maillard J.Y., Russell A.D. (2003). Aspects of the antimicrobial mechanisms of action of a polyquaternium and an amidoamine. J. Antimicrob. Chemother.

[16] Campanhã M.T.N., Mamizuka E.M., Carmona-Ribeiro A.M. (1999). Interactions between cationic liposomes and bacteria: The physical-chemistry of the bactericidal action. J. Lipid Res.

[17] Martins L.M.S., Mamizuka E.M., Carmona-Ribeiro A.M. (1997). Cationic vesicles as bactericides. Langmuir.

[18] Kuegler R., Bouloussa O., Rondelez F. (2005). Evidence of a charge-density threshold for optimum efficiency of biocidal cationic surfaces. Microbiology.

[19] Endo Y., Tani T., Kodama M. (1987). Antimicrobial activity of tertiary amine covalently bonded to a polystyrene fiber. Appl. Environ. Microbiol.

[20] Fidai S., Farer S.W., Hancock R.E. (1997). Interaction of cationic peptides with bacterial membranes. Methods Mol. Biol.

[21] Friedrich C.L., Moyles D., Beverige T.J., Hancock R.E. (2000). Antibacterial action of structurally diverse cationic peptides on gram-positive bacteria. Antimicrob. Agents Chemother.

[22] Isquith A.J., Abbott E.A., Walters P.A. (1972). Surface-bonded antimicrobial activity of an organosilicon quaternary ammonium chloride. Appl. Microbiol.

[23] Tapias G.N., Sicchierolli S.M., Mamizuka E.M., Carmona-Ribeiro A.M. (1994). Interactions between cationic vesicles and *Escherichia coli*. Langmuir.

[24] Sicchierolli S.M., Mamizuka E.M., Carmona-Ribeiro A.M. (1995). Bacteria flocculation and death by cationic vesicles. Langmuir.

[25] Campanhã M.T.N., Mamizuka E.M., Carmona-Ribeiro A.M. (2001). Interactions between cationic vesicles and *Candida albicans*. J. Phys. Chem. B.

[26] Lincopan N., Mamizuka E.M., Carmona-Ribeiro A.M. (2003). *In vivo* activity of a novel amphotericin B formulation with synthetic cationic bilayer fragments. J. Antimicrob. Chemother.

[27] Lincopan N., Mamizuka E.M., Carmona-Ribeiro A.M. (2005). Low nephrotoxicity of an effective amphotericin B formulation with cationic bilayer fragments. J. Antimicrob. Chemother.

[28] Thome J., Holländer A., Jaeger W., Trick I., Oehr C. (2003). Ultrathin antibacterial polyammonium coatings on polymer surfaces. Surf. Coat. Technol..

[29] Kanazawa A., Ikeda T., Endo T. (1993). Novel polycationic biocides: Synthesis and antibacterial activity of polymeric phosphonium salts. J. Polym. Sci. A.

[30] Popa A., Davidescu C.M., Trif R., Ilia G.H., Iliescu S., Dehelean G.H. (2003). Study of quaternary ‘onium’ salts grafted on polymers: Antibacterial activity of quaternary phosphonium salts grafted on ‘gel-type’ styene-divinylbenzene copolymers. React. Funct. Polym.

[31] Tiller J.C., Liao C., Lewis K., Klibanov A.M. (2001). Designing surfaces that kill bacteria on contact. Proc. Natl. Acad. Sci. USA.

[32] Vieira D.B., Lincopan N., Mamizuka E.M., Petri D.F.S., Carmona-Ribeiro A.M. (2003). Competitive adsorption of cationic bilaers and chitosan on latex: Optimal biocidal action. Langmuir.

[33] Cen L., Neoh K.G., Kang E.T. (2003). Surface functionalization technique for conferring antibacterial properties to polymeric and cellulosic surfaces. Langmuir.

[34] Chen C.Z., Cooper S.L. (2000). Recent advances in antimicrobial dendrimers. Adv. Mater.

[35] Chen C.Z., Cooper S.L. (2002). Interactions between dendrimer biocides and bacterial membranes. Biomaterials.

[36] Carmona-Ribeiro A.M., Chaimovich H. (1983). Preparation and characterization of large dioctadecyl dimethylammonium chloride liposomes and comparison with small sonicated vesicles. Biochim. Biophys. Acta.

[37] Carmona-Ribeiro A.M., Yoshida L.S., Chaimovich H. (1985). Salt effects on the stability of dioctadecyldimethylammonium chloride and sodium dihexadecyl phosphate vesicles. J. Phys. Chem.

[38] Carmona-Ribeiro A.M., Chaimovich H. (1986). Salt-induced aggregation and fusion of dioctadecyl dimethylammonium chloride and sodium dihexadecylphosphate vesicles. Biophys. J.

[39] Carmona-Ribeiro A.M. (1992). Synthetic amphiphile vesicles. Chem. Soc. Rev.

[40] Vieira D.B., Carmona-Ribeiro A.M. (2001). Synthetic bilayer fragments for solubilization of amphotericin B. J. Colloid Interface Sci.

[41] Carmona-Ribeiro A.M., Mukherjee A. (2010). Lipid-Based Biomimetics in Drug and Vaccine Delivery. Biomimetics Learning from Nature.

[42] Carmona-Ribeiro A.M., Barbassa L., Melo L.D., Cavrak M. (2011). Antimicrobial Biomimetics. Biomimetic Based Applications.

[43] Ahlstrom B., Chelminska-Bertilsson M., Thompson R.A., Edebo L. (1997). Submicellar complexes may initiate the fungicidal effects of cationic amphiphilic compounds on *Candida albicans*. Antimicrob. Agents Chemother.

[44] Vieira D.B., Carmona-Ribeiro A.M. (2006). Cationic lipids and surfactants as antifungal agents: Mode of action. J. Antimicrob. Chemother.

[45] Melo L.D., Mamizuka E.M., Carmona-Ribeiro A.M. (2010). Antimicrobial particles from cationic lipid and polyelectrolytes. Langmuir.

[46] Carmona-Ribeiro A.M., Midmore B.R. (1992). Synthetic bilayer adsorption onto polystyrene microspheres. Langmuir.

[47] Rosa H., Petri D.F.S., Carmona-Ribeiro A.M. (2008). Interactions between bacteriophage DNA and cationic biomimetic particles. J. Phys. Chem. B.

[48] Lambert P.A., Hammond S.M. (1973). Potassium fluxes, first indications of membrane damage in micro-organisms. Biochem. Biophys. Res. Commun.

[49] Menschutkin N. (1890). Beiträgen zur kenntnis der affinitätskoeffizienten der alkylhaloide und der organischen amine. Z. Phys. Chem.

[50] Jacobs W.A., Heidelberger M. (1915). The quaternary salts of hexamethylenetetramine. I. Substituted benzyl halides and the hexamethylenetetraminium salts derived therefrom. J. Biol. Chem.

[51] Jacobs W.A., Heidelberger M. (1915). The quaternary salts of hexamethylenetetramine. II. Monohalogenacetylbenzylamines and their hexamethylenetetraminium salts. J. Biol. Chem.

[52] Jacobs A.A., Heidelberger M. (1915). The quaternary salts of hexamethylenetetramine. III. monohalogenacylated aromatic amines and their hexamethylenetetraminium salts. J. Biol. Chem.

[53] Jacobs W.A. (1916). Tile bactericidal properties of the quaternary salts of hexamethylenetetramine. I. The problem of the chemotherapy of experimental bacterial infections. J. Exp. Med.

[54] Domagk G. (1935). A new class of disinfectant. Dtsch. Med. Wochenschr.

[55] Denmark S.E., Gould N.D., Wolf L.M. (2011). A systematic investigation of quaternary ammonium ions as asymmetric phase-transfer catalysts. Application of quantitative structure activity/selectivity relationships. J. Org. Chem.

[56] Butenschön H. (1995). Book Review: Organic syntheses based on name reactions and unnamed reaction. By Hassner, A., Stumer, C. Angew. Chem. Int. Ed. Engl.

[57] Butcher J.A., Preston A.F., Drysdale J. (1977). Initial screening trials of some quaternary ammonium compounds and amine salts as wood preservatives. Forest Product J.

[58] Butcher J.A., Drysdale J. (1977). Relative tolerance of 7 wood-destroying basidiomycetes to quaternary ammonium-compounds and copper-chrome-arsenate preservative. Mater. Organismen.

[59] Welton T. (1999). Room-temperature ionic liquids. Solvents for synthesis and catalysis. Chem. Rev.

[60] Wasserscheid P., Keim W. (2000). Ionic liquids—New “solutions” for transition metal catalysis. Angew. Chem. Int. Ed. Engl.

[61] Sheldon R. (2001). Catalytic reactions in ionic liquids. Chem. Commun.

[62] Olivier-Bourbigou H., Magna L. (2002). Ionic liquids: Perspectives for organic and catalytic reactions. J. Mol. Catal. A Chem..

[63] Dupont J., de Souza R.F., Suarez P.A.Z. (2002). Ionic liquid (molten salt) phase organometallic catalysis. Chem. Rev.

[64] Earle M.J., Rogers R.D., Seddon K.R. (2002). Ionic Liquids: Solvents for the Twenty-First Century. Ionic Liquids: Industrial Applications for Green Chemistry.

[65] Davis H., Gordon C.M., Hilgers C., Wasserscheid P., Wasserscheid P., Welton T. (2002). Synthesis and Purification of Ionic Liquids. Ionic Liquids in Synthesis.

[66] Sidhu M.S., Sorum H., Holck A. (2002). Resistance to quaternary ammonium compounds in food-related bacteria. Microb. Drug Resist.

[67] Carmona-Ribeiro A.M., Vieira D.B., Lincopan N. (2006). Cationic lipids and surfactants as anti-infective agents. Anti-Infect. Agents Med. Chem.

[68] Nishihara T., Okamoto T., Nishiyama N. (2000). Biodegradation of didecyldimethylammonium chloride by *Pseudomonas fluorescens* TN4 isolated from activated sludge. J. Appl. Microbiol.

[69] Arnt L., Tew G.N. (2002). New poly(phenyleneethynylene)s with cationic, facially amphiphilic structures. J. Am. Chem. Soc.

[70] Diaz T., Fischer A., Jonquières A., Brembilla A., Lochon P. (2003). Controlled polymerization of functional monomers and synthesis of block copolymers using beta-phosphonylated nitroxide. Macromolecules.

[71] Jaeger W., Wendler U., Lieske A., Bohrisch J. (1999). Novel modified polymers with permanent cationic groups. Langmuir.

[72] Oster C.G., Wittmar M., Unger F., Barbu-Tudoran L., Schaper A.K., Kissel T. (2004). Design of amine-modified graft polyesters for effective gene delivery using DNA-loaded nanoparticles. Pharm. Res.

[73] Tadros T. (2009). Polymeric surfactants in disperse systems. Adv. Colloid Interface Sci..

[74] Summers M., Eastoe J. (2003). Concentrated polymerized cationic surfactant phases. Langmuir.

[75] Chung M.-H., Park J.-H., Chun B.C., Chung Y.-C. (2004). Polymerizable ion-pair amphiphile that has a polymerizable group at cationic ammonium chain. Colloids Surf. B Biointerfaces.

[76] Liu S.Y., González Y.I., Kaler E.W. (2003). Structural fixation of spontaneous vesicles in aqueous mixtures of polymerizable anionic and cationic surfactants. Langmuir.

[77] Holmberg K. (2003). Novel Surfactants. Preparation, Applications, and Biodegradability.

[78] Gerber M.J., Kline S.R., Walker L.M. (2004). Characterization of rodlike aggregates generated from a cationic surfactant and a polymerizable counterion. Langmuir.

[79] Deen G.R., Gan L.H., Gan Y.Y. (2004). A new cationic surfactant *N,N′*-dimethyl-*N*-acryloyloxyundecyl piperazinium bromide and its pH-sensitive gels by microemulsion polymerization. Polymer.

[80] Zelikin A.N., Putnam D., Shastri P., Langer R., Izumrudov V.A. (2002). Aliphatic ionenes as gene delivery agents: Elucidation of structure-funtion relationship through modification of charge density and polymer length. Bioconjug. Chem.

[81] Izumrudov V.A., Zhiryakova M.V., Kudaibergenov S.E. (1999). Controllable stability of DNA-containing polyelectrolyte complexes in water-salt solutions. Biopolymers.

[82] Aubin R.A., Weinfeld M., Mirzayans R., Paterson M.C. (1994). Polybrene/DMSO-assisted gene transfer generating stable transfectants with nanogram amounts of DNA. Mol. Biotechnol.

[83] Rembaum A., Baumgartner W., Eisenberg A. (1968). Aliphatic ionenes. J. Polym. Sci. B.

[84] Littmann E.R., Marvel C.S. (1930). Cyclic quaternary ammonium salt from halogenated aliphatic tertiary amines. J. Am. Chem. Soc.

[85] Zelikin A.N., Akritskaya N.I., Izumrudov V.A. (2001). Modified aliphatic ionenes. Influence of charge density and length of the chains on complex formation with poly (methacrylic acid). Macromol. Chem. Phys.

[86] Ikeda T., Yamaguchi H., Tazuke S. (1990). Phase-separation in phospholipid bilayers induced by biologically-active polycations. Biochim. Biophys. Acta.

[87] Rembaum A., Senyei A.E., Rajaraman R. (1977). Interaction of living cells with polyionenes and polyionene-coated surfaces. J. Biomed. Mater. Res.

[88] Kimura E.T., Young P.R., Barlow G.H. (1962). Study with low and high molecular weights of hexadimethrine bromide—An antiheparin agent. Exp. Biol. Med.

[89] Putnam D., Gentry C.A., Pack D.W., Langer R. (2001). Polymer-based gene delivery with low cytotoxicity by a unique balance of side chain termini. Proc. Natl. Acad. Sci. USA.

[90] Wolfert M.A., Dash P.R., Nazarova O., Oupicky D., Seymour L.W., Smart S., Strhalm J., Ulbrich K. (1999). Polyelectrolyte vectors for gene delivery: Influence of cationic polymer on biophysical properties of complexes formed with DNA. Bioconjug. Chem.

[91] Ilker M.F., Schule H., Coughlin E.B. (2004). Modular norbornene derivatives for the preparation of well-defined amphiphilic polymers: Study of the lipid membrane disruptions activities. Macromolecules.

[92] Ilker M.F., Nuesslein K., Tew G.N., Coughlin E.B. (2004). Tuning the hemolytic and antibacterial activities of amphiphilic polynorbornene derivatives. J. Am. Chem. Soc.

[93] Kusnetsov J.M., Tulkki A.I., Ahonen H.E., Martikainen P.J. (1997). Efficacy of three prevention strategies against legionella in cooling water systems. J. Appl. Microbiol.

[94] Messick C.R., Pendland S.L., Moshirfar M., Fiscella R.G., Losnedahl K.J., Schriever C.A., Schreckenberger P.C. (1999). *In-vitro* activity of polyhexamethylene biguanide (PHMB) against fungal isolates associated with infective keratitis. J. Antimicrob. Chemother.

[95] Donoso R., Mura J.J., Lopez M. (2002). *Acanthamoeba* keratitis treated with propamidine and polyhexamethyl biguanide. Rev. Med. Chile.

[96] Gray T.B., Gross K.A., Cursons R.T.M., Shewan J.F. (1994). *Acanthamoeba*-keratitis—A sobering case and a promising new treatment. Aust. NZJ. Ophthalmol.

[97] Narasimhan S., Madhavan H.N., Therese L.K. (2002). Development and application of an *in vitro* susceptibility test for *Acanthamoeba* species isolated from keratitis to polyhexamethylene biguanide and chlorhexidine. Cornea.

[98] Hiti K., Walochnik J., Haller-Schober E.M., Faschinger C., Aspock H. (2002). Viability of *Acanthamoeba* after exposure to a multipurpose disinfecting contact lens solution and two hydrogen peroxide systems. Br. J. Ophthalmol.

[99] Rosin M., Welk A., Bernhardt O., Ruhnau M., Pitten F.A., Kocher T., Kramer A. (2001). Effect of a polyhexamethylene biguanide mouthrinse on bacterial counts and plaque. J. Clin. Periodontol.

[100] Rosin M., Welk A., Kocher T., Majic-Todt A., Kramer A., Pitten F.A. (2002). The effect of a polyhexamethylene biguanide mouthrinse compared to an essential oil rinse and a chlorhexidine rinse on bacterial counts and 4-day plaque regrowth. J. Clin. Periodontol.

[101] Payne J.D., Kudner D.W. (1996). A durable antiodor finish for cotton textiles. Text. Chem. Color.

[102] Davis S., Mertz P.M., Cazzaniga A., Serralta V., Orr R., Eaglstein W. (2002). The use of new antimicrobial gauze dressings: Effects on the rate of epithelization of partial-thickness wounds. Wounds.

[103] Cox N.A., Bailey J.S., Berrang M.E. (1998). Bactericidal treatment of hatching eggs I. Chemical immersion treatments and *Salmonella*. J. Appl. Poult. Res.

[104] Cox N.A., Berrang M.E., Buhr R.J., Bailey J.S. (1999). Bactericidal treatment of hatching eggs II. Use of chemical disinfectants with vaccum to reduce *Salmonella*. J. Appl. Poult. Res.

[105] Allen M.J., Morby A.P., White G.F. (2004). Cooperativity in the binding of the cationic biocide polyhexamethylene biguanide to nucleic acids. Biochem. Biophys. Res. Commun.

[106] Zhou Z., Wei D., Guan Y., Zheng A., Zhong J.-J. (2011). Extensive *in vitro* activity of guanidine hydrochloride polymer analogs against antibiotics-resistant clinically isolated strains. Mater. Sci. Eng. C.

[107] Tashiro T. (2001). Antibacterial and bacterium adsorbing macromolecules. Macromol. Mater. Eng.

[108] Kawabata N., Nishiguchi M. (1988). Antibacterial activity of soluble pyridinium-type polymers. Appl. Environ. Microbiol.

[109] Carmona-Ribeiro A.M. (2003). Bilayer-forming synthetic lipids: Drugs or carriers?. Curr. Med. Chem.

[110] Carmona-Ribeiro A.M., Ortis F., Schumacher R.I., Armelin M.C.S. (1997). Interactions between cationic vesicles and cultures mammalian cells. Langmuir.

[111] Eliyahu H., Barenholz Y., Domb A.J. (2005). Polymers for DNA delivery. Molecules.

[112] Tros de Ilarduya C., Sun Y., Düzgüneş N. (2010). Gene delivery by lipoplexes and polyplexes. Eur. J. Pharm. Sci.

[113] Niculescu-Duvaz D., Heyes J., Springer C.J. (2003). Structure-activity relationship in cationic lipid mediated gene transfection. Curr. Med. Chem.

[114] Pedroso de Lima M.C., Neves S., Filipe A., Duezgunes N., Simoes S. (2003). Cationic liposomes for gene delivery: From biophysics to biological applications. Curr. Med. Chem.

[115] Miller A.D. (2003). The problem with cationic liposome/micelle-based non-viral vector systems for gene therapy. Curr. Med. Chem.

[116] Safinya C.R., Lin A.J., Slack N.L., Koltover I., Mahato R.I., Kim S.W. (2002). Structure and Structure-Activity Correlations of Cationic Lipid/DNA Complexes: Supramolecular Assembly and Gene Delivery. Pharmaceutical Perspectives of Nucleic Acid-Based Therapeutics.

[117] Belmont P., Aissaoui A., Hauchecorne M., Oudrhiri N., Petit L., Vigneron J.-P., Lehn J.-M., Lehn P. (2002). Aminoglycoside-derived cationic lipids as efficient vectors for gene transfection *in vitro* and *in vivo*. J. Gene Med.

[118] Simberg D., Weisman S., Talmon Y., Barenholz Y. (2004). DOTAP (and other cationic lipids): Chemistry, biophysics and transfection. Crit. Rev. Ther. Drug Carrier Syst.

[119] Aissaoui A., Martin B., Kan E., Oudrhiri N., Hauchecorne M., Vigneron J.P., Lehn J.M., Lehn P. (2004). Novel cationic lipids incorporating an acid-sensitive acylhydrazone linker: Synthesis and transfection properties. J. Med. Chem.

[120] Mahidhar Y.V., Rajesh M., Chaudhuri A. (2004). Spacer-arm modulated gene delivery efficacy of novel cationic glycolipids: Design, synthesis and *in vitro* transfection biology. J. Med. Chem.

[121] Ilies M.A., Seitz W.A., Ghiviriga I., Johnson B.H., Miller A., Thompson E.B., Balaban A.T. (2004). Pyridinium cationic lipids in gene delivery: A strucuture-activity correlation study. J. Med. Chem.

[122] Byk G., Dubertret C., Escriou V., Frederic M., Jaslin G., Rangara R., Pitard B., Crouzet J., Wils P., Schwartz B. (1998). Synthesis, activity and structure-activity relationship studies of novel cationic lipids for DNA transfer. J. Med. Chem.

[123] Smisterova J., Wagenaar A., Stuart M.C.A., Polushkin E., ten Brinke G., Hulst R., Engberts J.B.F.N., Hoekstra D. (2001). Molecular shape of the cationic lipid controls the structure of cationic lipid/dioleylphosphatidylethanolamine-DNA complexes and the efficiency of gene delivery. J. Biol. Chem.

[124] van der Woude I., Wagenaar A., Meekel A.A.P., ter Beest M.B.A., Ruiters M.H.J., Engberts J.B.F.N., Hoekstra D. (1997). Novel pyridinium surfactants for efficient, nontoxic *in vitro* gene delivery. Proc. Natl. Acad. Sci. USA.

[125] Karmali P.P., Kumar V.V., Chaudhuri A. (2004). Design, synthesis and *in vitro* gene delivery efficacies of novel mono- and trilysinated cationic lipids: A structure-activity investigation. J. Med. Chem.

[126] Floch V., Loisel S., Guenin E., Hervé A.C., Clement J.C., Yaouanc J.J., des Abbayes H., Férec C. (2000). Cation substitution in cationic phosphonolipids: A new concept to improve transfection activity and decrease cellular toxicity. J. Med. Chem.

[127] Jacopin C., Ho H., Scherman D., Herscovici J. (2001). Synthesis and transfecting properties of a glycosylated polycationic DNA vector. Bioorg. Med. Chem. Lett.

[128] Vigneron J.P., Oudrhiri N., Fauquet M., Vergely L., Bradley J.C., Basseville M., Lehn P., Lehn J.M. (1996). Guanidinium-cholesterol cationic lipids: Efficient vectors for the transfection of eukaryotic cells. Proc. Natl. Acad. Sci. USA.

[129] Patel M., Vivien E., Hauchecorne M., Oudrhiri N., Ramasawmy R., Vigneron J.P., Lehn P., Lehn J.M. (2001). Efficient gene transfection by bisguanylated diacetylene lipid formulations. Biochem. Biophys. Res. Commun.

[130] Marshall J., Nietupski J.B., Lee E.R., Siegel C.S., Rafter P.W., Rudginsky S.A., Chang C.D., Eastman S.J., Harris D.J., Scheule R.K. (2000). Cationic lipid structure and formulation considerations for optimal gene transfection of the lung. J. Drug Target.

[131] Layman J.M., Ramirez S.M., Green M.D., Long T.E. (2009). Influence of polycation molecular weight on poly (2-dimethylaminoethyl methacrylate)-mediated DNA delivery *in vitro*. Biomacromolecules.

[132] Matyjaszewski K., Davis T.P. (2002). Handbook of Radical Polymerization.

[133] Venkataraman S., Zhang Y., Liu L., Yang Y.-Y. (2010). Design, syntheses and evaluation of hemocompatible pegylated-antimicrobial polymers with well-controlled molecular structures. Biomaterials.

[134] Ji W., Panus D., Palumbo R.N., Tang R., Wang C. (2011). Poly (2-aminoethyl methacrylate) with well-defined chain length for DNA vaccine delivery to dendritic cells. Biomacromolecules.

[135] Kuroda K., DeGrado W.F. (2005). Amphiphilic polymethacrylate derivatives as antimicrobial agents. J. Am. Chem. Soc.

[136] Feder R., Dagan A., Mor A. (2000). Structure-activity relationship study of antimicrobial dermaseptin S4 showing the consequences of peptide oligomerization on selective cytotoxicity. J. Biol. Chem.

[137] Palermo E.F., Kuroda K. (2010). Structural determinants of antimicrobial activity in polymers which mimic host defense peptides. Appl. Microbiol. Biotechnol.

[138] Hamuro Y., Schneider J.P., DeGrado W.F. (1999). *De novo* design of antibacterial beta-peptides. J. Am. Chem. Soc.

[139] Patch J.A., Barron A.E. (2003). Helical peptoid mimics of magainin-2 amide. J. Am. Chem. Soc.

[140] Sellenet P.H., Allison B., Applegate B.M., Youngblood J.P. (2007). Synergistic activity of hydrophilic modification in antibiotic polymers. Biomacromolecules.

[141] Kuroda K., Caputo G.A., DeGrado W.F. (2009). The role of hydrophobicity in the antimicrobial and hemolytic activities of polymethacrylate derivatives. Chemistry.

[142] Mowery B.P., Lee S.E., Kissounko D.A., Epand R.F., Epand R.M., Weisblum B., Stahl S.S., Gellman S.H. (2007). Mimicry of antimicrobial host-defense peptides by random copolymers. J. Am. Chem. Soc.

[143] Palermo E.F., Lee D.-K., Ramamoorthy A., Kuroda K. (2011). Role of cationic group structure in membrane binding and disruption by amphiphilic copolymers. J. Phys. Chem. B.

[144] Azzam T., Domb A.J. (2004). Current developments in gene transfection agents. Curr. Drug Deliv.

[145] Khalil H., Chen T., Riffon R., Wang R., Wang Z. (2008). Synergy between polyethylenimine and different families of antibiotics against a resistant clinical isolate of *Pseudomonas aeruginosa*. Antimicrob. Agents Chemother.

[146] Fuchs B.B., Tegos G.P., Hamblin M.R., Mylonakis E. (2007). Susceptibility of *Cryptococcus neoformans* to photodynamic inactivation is associated with cell wall integrity. Antimicrob. Agents Chemother.

[147] Tegos G.P., Anbe M., Yang C., Demidova T.N., Satti M., Mroz P., Janjua S., Gad F., Hamblin M.R. (2006). Protease-stable polycationic photosensitizer conjugates between polyethyleneimine and chlorin(e6) for broad-spectrum antimicrobial photoinactivation. Antimicrob. Agents Chemother.

[148] Milovic N.M., Wang J., Lewis K., Klibanov A.M. (2005). Immobilized N-alkylated polyethylenimine avidly kills bacteria by rupturing cell membranes with no resistance developed. Biotechnol. Bioeng.

[149] Spoden G.A., Besold K., Krauter S., Plachter B., Hanik N., Kilbinger A.F., Lambert C., Florin L. (2012). Polyethylenimine is a strong inhibitor of human papillomavirus and cytomegalovirus infection. Antimicrob. Agents Chemother.

[150] Soukos N.S., Ximenez-Fyvie L.A., Hamblin M.R., Socransky S.S., Hasan T. (1998). Targeted antimicrobial photochemotherapy. Antimicrob. Agents Chemother.

[151] Sharma S.K., Dai T., Kharkwal G.B., Huang Y.Y., Huang L., Bil De Arce V.J., Tegos G.P., Hamblin M.R. (2011). Drug discovery of antimicrobial photosensitizers using animal models. Curr. Pharm. Des.

[152] Lu L., Rininsl F.H., Wittenburg S.K., Achyuthan K.E., McBranch D.W., Whitten D.G. (2005). Biocidal activity of a light-absorbing fluorescent conjugated polyelectrolyte. Langmuir.

[153] Chemburu S., Corbitt T.S., Ista L.K., Ji E., Fulghum J., Lopez G.P., Ogawa K., Schanze K.S., Whitten D.G. (2008). Light-induced biocidal action of conjugated polyelectrolytes supported on colloids. Langmuir.

[154] Tang Y.L., Zhou Z.J., Ogawa K., Lopez G.P., Schanze K.S., Whitten D.G. (2009). Synthesis, self-assembly, and photophysical behavior of oligo phenylene ethynylenes: From molecular to supramolecular properties. Langmuir.

[155] Tang Y.L., Hill E.H., Zhou Z.J., Evans D.G., Schanze K.S., Whitten D.G. (2011). Synthesis, self-assembly, and photophysical properties of cationic oligo(p-phenyleneethynylene)s. Langmuir.

[156] Zhou Z.J., Corbitt T.S., Parthasarathy A., Tang Y.L., Ista L.F., Schanze K.S., Whitten D.G. (2010). “End-only” functionalized oligo(phenylene ethynylene)s: Synthesis, photophysical and biocidal activity. J. Phys. Chem. Lett.

[157] Corbitt T.S., Sommer J.R., Chemburu S., Ogawa K., Ista L.K., Lopez G.P., Whitten D.G., Schanze K.S. (2009). Conjugated polyelectrolyte capsules: Light-activated antimicrobial micro “Roach Motels”. ACS Appl. Mater. Interfaces.

[158] Zhao X.Y., Pinto M.R., Hardison L.M., Mwaura J., Muller J., Jiang H., Witker D., Kleiman V.D., Reynolds J.R., Schanze K.S. (2006). Variable band gap poly (arylene ethynylene) conjugated polyelectrolytes. Macromolecules.

[159] Wang Y., Zhou Z.J., Zhu J.S., Tang Y.L., Canady T.D., Chi E.Y., Schanze K.S., Whitten D.G. (2011). Dark antimicrobial mechanisms of cationic phenylene ethynylene polymers and oligomers against *Escherichia coli*. Polymers.

[160] Lee H., Larson R.G. (2008). Lipid bilayer curvature and pore formation induced by charged linear polymers and dendrimers: The effect of molecular shape. J. Phys. Chem. B.

[161] Melo M.N., Ferre R., Castanho M.A.R.B. (2009). Antimicrobial peptides: Linking partition, activity and high membrane-bound concentrations. Nat. Rev. Microbiol.

[162] Zasloff M. (2002). Antimicrobial peptides of multicellular organisms. Nature.

[163] Brogden K.A. (2005). Antimicrobial peptides: Pore formers or metabolic inhibitors in bacteria?. Nat. Rev. Microbiol.

[164] Pereira A.H. (2006). Novel therapeutics based on cationic peptides. Curr. Pharm. Biotechnol.

[165] Rossi L.M., Rangasamy P., Zhang J., Qui X.-Q., Wu G.Y. (2008). Research advances in the development of peptides antibiotics. J. Pharm. Sci.

[166] Devine D.A., Hancock R.E.W. (2002). Cationic peptides: Distribution and mechanisms of resistance. Curr. Pharm. Des.

[167] Song Y.M., Park Y., Lim S.S., Yang S.T., Woo E.R., Park S., Lee J.S., Kim J.I., Hahm K.S., Kim Y. (2005). Cell selectivity and mechanism of action of antimicrobial model peptides containing peptoid residues. Biochemistry.

[168] Fimland G., Johnsen L., Dalhus B., Nissen-Meyer J. (2005). Pediocin-like antimicrobial peptides (class IIa bacteriocins) and their immunity proteins: Biosynthesis, structure, and mode of action. J. Pept. Sci.

[169] Hancock R.E.W., Lehrer R. (1998). Cationic peptides: A new source of antibiotics. Trends Biotechnol.

[170] Toke O. (2005). Antimicrobial peptides: New candidates in the fight against bacterial infections. Biopolymers.

[171] Hancock R.E., Sahl H.G. (2006). Antimicrobial and host-defense peptides as new anti-infective therapeutic strategies. Nat. Biotechnol.

[172] McAuliffe O., Ross R.P., Hill C. (2001). Lantibiotics: Structure, biosynthesis and mode of action. FEMS Microbiol. Rev.

[173] Hsu S.T., Breukink E., Tischenko E., Lutters M.A., Kruijff B., Kaptein R., Bonvin A.M., van Nuland N.A. (2004). The nisin-lipid II complex reveals a pyrophosphate cage that provides a blueprint for novel antibiotics. Nat. Struct. Mol. Biol.

[174] Perlman D., Bodanszky M. (1971). Biosynthesis of peptide antibiotics. Annu. Rev. Biochem.

[175] Kleinkauf H., von Dohren H. (1988). Peptide antibiotics, β-lactams and related compounds. Crit. Rev. Biotechnol.

[176] Hancock R.E., Falla T., Brown M.H. (1995). Cationic bactericidal peptides. Adv. Microb. Physiol.

[177] Weidenmaier C., Kristian S.A., Peschel A. (2003). Bacterial resistance to antimicrobial host defenses—An emerging target for novel antiinfective strategies?. Curr. Drug Targets.

[178] Koprivnjak T., Peschel A. (2011). Bacterial resistance mechanisms against host defense peptides. Cell. Mol. Life Sci.

[179] Saar-Dover R., Bitler A., Nezer R., Shmuel-Galia L., Firon A., Shimoni E., Trieu-Cuot P., Shai Y. (2012). D-alanylation of lipoteichoic acids confers resistance to cationic peptides in group B streptococcus by increasing the cell wall density. PLoS Pathog.

[180] Palusińska-Szysz M., Zdybicka-Barabas A., Pawlikowska-Pawlęga B., Mak P., Cytryńska M. (2012). Anti-*Legionella dumoffii* activity of galleria mellonella defensin and apolipophorin III. Int. J. Mol. Sci.

[181] Papagianni M. (2003). Ribosomally synthesized peptides with antimicrobial properties: Biosynthesis, structure, function, and applications. Biotechnol. Adv.

[182] Cotter P.D., Hill C., Ross R.P. (2005). Bacteriocins: Developing innate immunity for food. Nat. Rev. Microbiol.

[183] Dufour A., Hindré T., Haras D., Le Pennec J.P. (2007). The biology of lantibiotics from the lacticin 481 group is coming of age. FEMS Microbiol. Rev.

[184] Gardiner G.E., Rea M.C., O'Riordan B., O'Connor P., Morgan S.M., Lawlor P.G., Lynch P.B., Cronin M., Ross R.P., Hill C. (2007). Fate of the two-component lantibiotic lacticin 3147 in the gastrointestinal tract. Appl. Environ. Microbiol.

[185] Svenson J., Brandsdal B.O., Stensen W., Svendsen J.S. (2007). Albumin binding of short cationic antimicrobial micropeptides and its influence on the *in vitro* bactericidal effect. J. Med. Chem.

[186] Svenson J., Stensen W., Brandsdal B.O., Haug B.E., Monrad J., Svendsen J.S. (2008). Antimicrobial peptides with stability toward tryptic degradation. Biochemistry.

[187] Makovitzki A., Shai Y. (2005). pH-dependent antifungal lipopeptides and their plausible mode of action. Biochemistry.

[188] Chongsiriwatana N.P., Miller T.M., Wetzler M., Vakulenko S., Karlsson A.J., Palecek S.P., Mobashery S., Barron A.E. (2011). Short alkylated peptoid mimics of antimicrobial lipopeptides. Antimicrob. Agents Chemother.

[189] Chongsiriwatana N.P., Barron A.E. (2010). Comparing bacterial membrane interactions of antimicrobial peptides and their mimics. Methods Mol. Biol.

[190] Findlay B., Szelemej P., Zhanel G.G., Schweizer F. (2012). Guanidylation and tail effects in cationic antimicrobial lipopeptoids. PLoS One.

[191] Findlay B., Zhanel G.G., Schweizer F. (2012). Investigating the antimicrobial peptide “Window of activity” using cationic lipopeptides with hydrocarbon and fluorinated tails. Int. J. Antimicrob. Agents.

[192] Munoz-Bonilla A., Fernandez-Garcia M. (2012). Polymeric materials with antimicrobial activity. Prog. Polym. Sci.

[193] Moreira Mdel R., Pereda M., Marcovich N.E., Roura S.I. (2011). Antimicrobial effectiveness of bioactive packaging materials from edible chitosan and casein polymers: Assessment on carrot, cheese, and salami. J. Food Sci.

[194] Kuroda K., Caputo G.A. (2013). Antimicrobial polymers as synthetic mimics of host-defense peptides. Wiley Interdiscip. Rev. Nanomed. Nanobiotechnol.

[195] Timofeeva L., Kleshcheva N. (2011). Antimicrobial polymers: Mechanism of action, factors of activity, and applications. Appl. Microbiol. Biotechnol.

[196] Kenawy E-R., Worley S.D., Broughton R. (2007). The chemistry and applications of antimicrobial polymers: A state-of-the-art review. Biomacromolecules.

[197] Renani H.B., Ghorbani M., Beni B.H., Karimi Z., Mirhosseini M., Zarkesh H., Kabiri A. (2012). Determination and comparison of specifics of nucleus pulposus cells of human intervertebral disc in alginate and chitosan-gelatin scaffolds. Adv. Biomed. Res..

[198] Totonelli G., Maghsoudlou P., Fishman J.M., Orlando G., Ansari T., Sibbons P., Birchall M.A., Pierro A., Eaton S., de Coppi P. (2012). Esophageal tissue engineering: A new approach for esophageal replacement. World J. Gastroenterol.

[199] Hickok N.J., Shapiro I.M. (2012). Immobilized antibiotics to prevent orthopaedic implant infections. Adv. Drug Deliv. Rev.

[200] Dai T., Tanaka M., Huang Y.Y., Hamblin M.R. (2011). Chitosan preparations for wounds and burns: Antimicrobial and wound-healing effects. Expert Rev. Anti-Infect. Ther.

[201] Bitar K.N., Raghavan S. (2012). Intestinal tissue engineering: Current concepts and future vision of regenerative medicine in the gut. Neurogastroenterol. Motil.

[202] Walker P.A., Aroom K.R., Jimenez F., Shah S.K., Harting M.T., Gill B.S., Cox C.S. (2009). Advances in progenitor cell therapy using scaffolding constructs for central nervous system injury. Stem Cell Rev.

[203] Zhang Z., Hu J., Ma P.X. (2012). Nanofiber-based delivery of bioactive agents and stem cells to bone sites. Adv. Drug Deliv. Rev.

[204] Tan H., Ma R., Lin C., Liu Z., Tang T. (2013). Quaternized chitosan as an antimicrobial agent: Antimicrobial activity, mechanism of action and biomedical applications in orthopedics. Int. J. Mol. Sci.

[205] Valencia-Chamorro S.A., Palou L., Del Río M.A., Pérez-Gago M.B. (2011). Antimicrobial edible films and coatings for fresh and minimally processed fruits and vegetables: A review. Crit. Rev. Food Sci. Nutr.

[206] Cagri A., Ustunol Z., Ryser E.T. (2004). Antimicrobial edible films and coatings. J. Food Prot.

[207] Vargas M., González-Martínez C. (2010). Recent patents on food applications of chitosan. Recent Pat. Food Nutr. Agric.

[208] Francolini I., Donelli G. (2010). Prevention and control of biofilm-based medical-device-related infections. FEMS Immunol. Med. Microbiol.

[209] Bridier A., Sanchez-Vizuete Mdel P., Le Coq D., Aymerich S., Meylheuc T., Maillard J.Y., Thomas V., Dubois-Brissonnet F., Briandet R. (2012). Biofilms of a *Bacillus subtilis* hospital isolate protect *Staphylococcus aureus* from biocide action. PLoS One.

[210] Thallinger B., Prasetyo E.N., Nyanhongo G.S., Guebitz G.M. (2013). Antimicrobial enzymes: An emerging strategy to fight microbes and microbial biofilms. Biotechnol. J.

[211] Caro A., Humblot V., Méthivier C., Minier M., Salmain M., Pradier C.M. (2009). Grafting of lysozyme and/or poly(ethylene glycol) to prevent biofilm growth on stainless steel surfaces. J. Phys. Chem. B.

[212] Shukla A., Fleming K.E., Chuang H.F., Chau T.M., Loose C.R., Stephanopoulos G.N., Hammond P.T. (2010). Controlling the release of peptide antimicrobial agents from surfaces. Biomaterials.

[213] Melo L.D., Carmona-Ribeiro A.M. (2012). Fungicidal nanoparticles of low toxicity from cationic lipid and polyelectrolytes. NSTI Nanotech.

[214] Pereira E.M., Kosaka P.M., Rosa H., Vieira D.B., Kawano Y., Petri D.F., Carmona-Ribeiro A.M. (2008). Hybrid materials from intermolecular associations between cationic lipid and polymers. J. Phys. Chem. B.

[215] Dvoracek C.M., Sukhonosova G., Benedik M.J., Grunlan J.C. (2009). Antimicrobial behavior of polyelectrolyte-surfactant thin film assemblies. Langmuir.

[216] Melo L.D., Palombo R.R., Petri D.F.S., Bruns M., Pereira E.M.A., Carmona-Ribeiro A.M. (2011). Structure-activity relationship for quaternary ammonium compounds hybridized with poly(methyl methacrylate). ACS Appl. Mater. Interfaces.

[217] Decher G., Hong J.D. (1991). Buildup of ultrathin multilayer films by a self-assembly process: II. Consecutive adsorption of anionic and cationic bipolar amphiphiles and polyelectrolytes on charged surfaces. Ber. Bunsenges. Phys. Chem.

[218] Decher G. (1997). Fuzzy nanoassemblies: Toward layered polymeric multicomposites. Science.

[219] Song J., Kang H., Lee C., Hwang S.H., Jang J. (2012). Aqueous synthesis of silver nanoparticle embedded cationic polymer nanofibers and their antibacterial activity. ACS Appl. Mater. Interfaces.

[220] Fu J., Ji J., Yuan W., Shen J. (2005). Construction of anti-adhesive and antibacterial multilayer films via layer-by-layer assembly of heparin and chitosan. Biomaterials.

[221] Fu J., Ji J., Fan D., Shen J. (2006). Construction of antibacterial multilayer films containing nanosilver via layer-by-layer assembly of heparin and chitosan-silver ions complex. J. Biomed. Mater. Res. A.

[222] Cao H., Liu X. (2010). Silver nanoparticles-modified films *versus* biomedical device-associated infections. Wiley Interdiscip. Rev. Nanomed. Nanobiotechnol.

[223] Vieira D.B., Carmona-Ribeiro A.M. (2008). Cationic nanoparticles for delivery of amphotericin B: Preparation, characterization and activity *in vitro*. J. Nanobiotechnol..

[224] Zhang L., Eisenberg A. (1995). Multiple morphologies of “Crew-Cut” aggregates of polystyrene-b-poly( acrylic acid) block copolymers. Science.

[225] Discher D.E., Eisenberg A. (2002). Polymer vesicles. Science.

[226] Du J.Z., Chen Y.M. (2004). Organic-inorganic hybrid nanoparticles with a complex hollow structure. Angew. Chem. Int. Ed.

[227] Du J.Z., Willcock H., Ieong N.S., O'Reilly R.K. (2011). pH-responsive chiral nanostructures. Aust. J. Chem.

[228] Du J.Z., Armes S.P. (2008). Preparation of primary amine-based block copolymer vesicles by direct dissolution in water and subsequent stabilization by sol-gel chemistry. Langmuir.

[229] Du J.Z., Armes S.P. (2009). Preparation of biocompatible zwitterionic block copolymer vesicles by direct dissolution in water and subsequent silicification within their membranes. Langmuir.

[230] Seyfriedsberger G., Rametsteiner K., Kern W. (2006). Polyethylene compounds with antimicrobial surface properties. Eur. Polym. J.

[231] Ignatova M., Voccia S., Gilbert B., Markova N., Cossement D., Gouttebaron R., Jérôme R., Jérôme C. (2006). Combination of electrografting and atom-transfer radical polymerization for making the stainless steel surface antibacterial and protein antiadhesive. Langmuir.

[232] Jiang X., Zhang G., Narain R., Liu S. (2009). Fabrication of two types of shell-cross-linked micelles with “inverted” structures in aqueous solution from schizophrenic water-soluble ABC triblock copolymer via click chemistry. Langmuir.

[233] Lutz J-F. (2011). Thermo-switchable materials prepared using the OEGMA-platform. Adv. Mater.

[234] Lutz J.F., Hoth A. (2006). Preparation of ideal PEG analogues with a tunable thermosensitivity by controlled radical copolymerization of 2-(2-methoxyethoxy)ethyl methacrylate and oligo(ethylene glycol) methacrylate. Macromolecules.

[235] Zhang C., Zhu Y., Zhou C., Yuan W., Du J. (2013). Antibacterial vesicles by direct dissolution of a block copolymer in water. Polym. Chem.

[236] Dandekar A.M., Gouran H., Ibáñez A.M., Uratsu S.L., Agüero C.B., McFarland S., Borhani Y., Feldstein P.A., Bruening G., Nascimento R. (2012). An engineered innate immune defense protects grapevines from Pierce disease. Proc. Natl. Acad. Sci. USA.

[237] Kiss E., Heine E.T., Hill K., He Y.C., Keusgen N., Pénzes C.B., Schnöller D., Gyulai G., Mendrek A., Keul H. (2012). Membrane affinity and antibacterial properties of cationic polyelectrolytes with different hydrophobicity. Macromol. Biosci.

[238] Kusumo A., Bombalski L., Lin Q., Matyjaszewski K., Schneider J.W., Tilton R.D. (2007). High capacity, charge-selective protein uptake by polyelectrolyte brushes. Langmuir.

[239] Lee S.B., Koepsel R.R., Morley S.W., Matyjaszewski K., Sun Y., Russell A.J. (2004). Permanent, nonleaching antibacterial surfaces. 1. Synthesis by atom transfer radical polymerization. Biomacromolecules.

[240] Roy D., Knapp J.S., Guthrie J.T., Perrier S. (2008). Antibacterial cellulose fiber via RAFT surface graft polymerization. Biomacromolecules.

[241] Huang J., Murata H., Koepsel R.R., Russell A.J., Matyjaszewski K. (2007). Antibacterial polypropylene via surface-initiated atom transfer radical polymerization. Biomacromolecules.

[242] Rawlinson L.A., Ryan S.M., Mantovani G., Syrett J.A., Haddleton D.M., Brayden D.J. (2010). Antibacterial effects of poly(2-(dimethylamino ethyl)methacrylate) against selected gram-positive and gram-negative bacteria. Biomacromolecules.

[243] Dong H., Huang J., Koepsel R.R., Ye P., Russell A.J., Matyjaszewski K. (2011). Recyclable antibacterial magnetic nanoparticles grafted with quaternized poly(2-(dimethylamino)ethyl methacrylate) brushes. Biomacromolecules.

[244] Wang J., Yao K., Korich A.L., Li S., Ma S., Ploehn H.J., Iovine P.M., Wang C., Chu F., Tang C. (2011). Combining renewable gum rosin and lignin: Towards hydrophobic polymer composites by controlled polymerization. J. Polym. Sci. A.

[245] Yancheva E., Paneva D., Maximova V., Mespouille L., Dubois P., Manolova N., Rashkov I. (2007). Polyelectrolyte complexes between (cross-linked) *N*-carboxyethylchitosan and (quaternized) poly[2-(dimethylamino)ethyl methacrylate]: Preparation, characterization, and antibacterial properties. Biomacromolecules.

[246] Chen Y., Wilbon P.A., Chen Y.P., Zhou J., Nagarkatti M., Wang C., Chu F., Decho A.W., Tang C. (2012). Amphipathic antibacterial agents using cationic methacrylic polymers with natural rosin as pendant group. RSC Adv.

[247] Bucki R., Sostarecz A.G., Byfield F.J., Savage P.B., Janmey P.A. (2007). Resistance of the antibacterial agent ceragenin CSA-13 to inactivation by DNA of F-actin, and its activity in cystic fibrosis sputum. J. Antimicrob. Chemother.

[248] Bucki R., Namiot D.B., Namiot Z., Savage P.B., Janmey P.A. (2008). Salivary mucins inhibit antibacterial activity of cathelicidin-derived LL-37 peptide but not the cationic steroid CSA-13. J. Antimicrob. Chemother.

[249] Epand R.F., Pollard J.E., Wright J.O., Savage P.B., Epand R.M. (2010). Depolarization, bacterial membrane composition, and the antimicrobial action of ceragenins. Antimicrob. Agents Chemother.

[250] Lewis K. (2007). Persister cells, dormancy and infectious disease. Nat. Rev. Microbiol.

[251] Coates A.R., Hu Y. (2008). Targeting non-multiplying organisms as a way to develop novel antimicrobials. Trends Pharmacol. Sci.

[252] Hurdle J.G., O'Neill A.J., Chopra I., Lee R.E. (2011). Targeting bacterial membrane function: An underexploited mechanism for treating persistent infections. Nat. Rev. Microbiol.

[253] Barry C.E., Boshoff H.I., Dartois V., Dick T., Ehrt S., Flynn J., Schnappinger D., Wilkinson R.J., Young D. (2009). The spectrum of latent tuberculosis: Rethinking the biology and intervention strategies. Nat. Rev. Microbiol.

[254] Sinclair K.D., Pham T.X., Farnsworth R.W., Williams D.L., Loc-Carrillo C., Horne L.A., Ingebretsen S.H., Bloebaum R.D. (2012). Development of a broad spectrum polymer-released antimicrobial coating for the prevention of resistant strain bacterial infections. J. Biomed. Mater. Res. A.

[255] Green J.B., Fulghum T., Nordhaus M.A. (2011). Review of immobilized antimicrobial agents and methods for testing. Biointerphases.

[256] Engler A.C., Shukla A., Puranam S., Buss H.G., Jreige N., Hammond P.T. (2011). Effects of side group functionality and molecular weight on the activity of synthetic antimicrobial polypeptides. Biomacromolecules.

[257] Radzishevsky I.S., Rotem S., Bourdetsky D., Navon-Venezia S., Carmeli Y., Mor A. (2007). Improved antimicrobial peptides based on acyl-lysine oligomers. Nat. Biotechnol.

[258] Tew G.N., Scott R.W., Klein M.L., Degrado W.F. (2010). *De novo* design of antimicrobial polymers, foldamers, and small molecules: from discovery to practical applications. Acc. Chem. Res.

[259] Sovadinova I., Palermo E.F., Huang R., Thoma L.M., Kuroda K. (2011). Mechanism of polymer-induced hemolysis: Nanosized pore formation and osmotic lysis. Biomacromolecules.

[260] Stratton T.R., Applegate B.M., Youngblood J.P. (2011). Effect of steric hindrance on the properties of antibacterial and biocompatible copolymers. Biomacromolecules.

[261] Lienkamp K., Madkour A.E., Musante A., Nelson C.F., Nüsslein K., Tew G.N. (2008). Antimicrobial polymers prepared by ROMP with unprecedented selectivity: A molecular construction kit approach. J. Am. Chem. Soc.

[262] Li P., Poon Y.F., Li W., Zhu H.Y., Yeap S.H., Cao Y., Qi X., Zhou C., Lamrani M., Beuerman R.W. (2011). A polycationic antimicrobial and biocompatible hydrogel with microbe membrane suctioning ability. Nat. Mater.

[263] Li P., Zhou C., Rayatpisheh S., Ye K., Poon Y.F., Hammond P.T., Duan H., Chan-Park M.B. (2012). Cationic peptidopolysaccharides show excellent broad-spectrum antimicrobial activities and high selectivity. Adv. Mater.

[264] Wang R., Wang L., Zhou L., Su Y., Qiu F., Wang D., Wu J., Zhu X., Yan D. (2012). The effect of a branched architecture on the antimicrobial activity of poly(sulfone amines) and poly(sulfone amine)/silver nanocomposites. J. Mater. Chem.

[265] Fox M.E., Szoka F.C., Fréchet J.M. (2009). Soluble polymer carriers for the treatment of cancer: The importance of molecular architecture. Acc. Chem. Res.

[266] Oikonomou E.K., Bethani A., Bokias G., Kallitsis J.K. (2011). Poly (sodium styrene sulfonate)-b-poly( methyl methacrylate) diblock copolymers through direct atom transfer radical polymerization: Influence of hydrophilic-hydrophobic balance on self-organization in aqueous solution. Eur. Polym. J.

[267] Song J., Kong H., Jang J. (2009). Enhanced antibacterial performance of cationic polymer modified silica nanoparticles. Chem. Commun. (Camb.).

[268] Nigmatullin R., Gao F., Konovalova V. (2009). Permanent, non-leaching antimicrobial polyamide nanocomposites based on organoclays modified with a cationic polymer. Macromol. Mater. Eng.

[269] Qian L., Guan Y., Ziaee Z., He B., Zheng A., Xiao H. (2009). Rendering cellulose fibers antimicrobial using cationic β-cyclodextrin-based polymers included with antibiotics. Cellulose.

[270] Pan Y., Xiao H., Zhao G., He B. (2008). Antimicrobial and thermal-responsive layer-by-layer assembly based on ionic-modified guanidine polymer and PVA. Polym. Bull.

[271] Pfaffenroth C., Winkel A., Dempwolf W., Gamble L.J., Castner D.G., Stiesch M., Menzel H. (2011). Self-assembled antimicrobial and biocompatible copolymer films on titanium. Macromol. Biosci.

[272] Malmsten M. (2011). Antimicrobial and antiviral hydrogels. Soft Matter.

[273] Llorens A., Lloret E., Picouet P.A., Trbojevich R., Fernandez A. (2012). Metallic-based micro and nanocomposites in food contact materials and active food packaging. Trends Food Sci. Technol.

[274] Seil J.T., Webster T.J. (2012). Antimicrobial applications of nanotechnology: Methods and literature. Int. J. Nanomedicine.

[275] Li Y., Hindi K., Watts K.M., Taylor J.B., Zhang K., Li Z., Hunstad D.A., Cannon C.L., Youngs W.J., Wooley K.L. (2010). Shell crosslinked nanoparticles carrying silver antimicrobials as therapeutics. Chem. Commun. (Camb.).

[276] Ran J., Bénistant G., Campagne C., Périchaud A., Chai F., Blanchemain N., Perwuelz A. (2012). Characterization of nonwoven poly(ethylene terephtalate) devices functionalized with cationic polymer. J. Appl. Polym. Sci.

[277] Pan Y., Xiao H. (2011). Rendering rayon fibres antimicrobial and thermal-responsive via layer-by-layer self-assembly of functional polymers. Adv. Mater. Res..

[278] Kemp M.M., Kumar A., Clement D., Ajayan P., Mousa S., Linhardt R.J. (2009). Hyaluronan- and heparin-reduced silver nanoparticles with antimicrobial properties. Nanomedicine (Lond.).

[279] Chen J., Hessler J.A., Putchakayala K., Panama B.K., Khan D.P., Hong S., Mullen D.G., Dimaggio S.C., Som A., Tew G.N. (2009). Cationic nanoparticles induce nanoscale disruption in living cell plasma membranes. J. Phys. Chem. B.

[280] Leroueil P.R., Berry S.A., Duthie K., Han G., Rotello V.M., McNerny D.Q., Baker J.R., Orr B.G., Holl M.M. (2008). Wide varieties of cationic nanoparticles induce defects in supported lipid bilayers. Nano Lett.

[281] Epand R.F., Mowery B.P., Lee S.E., Stahl S.S., Lehrer R.I., Gellman S.H., Epand R.M. (2008). Dual mechanism of bacterial lethality for a cationic sequence-random copolymer that mimics host-defense antimicrobial peptides. J. Mol. Biol.

[282] Fröhlich E. (2012). The role of surface charge in cellular uptake and cytotoxicity of medical nanoparticles. Int. J. Nanomed.

[283] Rawlinson L.A., O'Gara J.P., Jones D.S., Brayden D.J. (2011). Resistance of *Staphylococcus aureus* to the cationic antimicrobial agent poly(2-(dimethylamino ethyl)methacrylate) (pDMAEMA) is influenced by cell-surface charge and hydrophobicity. J. Med. Microbiol.

[284] Rawlinson L.A., O'Brien P.J., Brayden D.J. (2010). High content analysis of cytotoxic effects of pDMAEMA on human intestinal epithelial and monocyte cultures. J. Control. Release.

[285] Nederberg F., Zhang Y., Tan J.P., Xu K., Wang H., Yang C., Gao S., Guo X.D., Fukushima K., Li L. (2011). Biodegradable nanostructures with selective lysis of microbial membranes. Nat. Chem.

[286] Nitta S.K., Numata K. (2013). Biopolymer-based nanoparticles for drug/gene delivery and tissue engineering. Int. J. Mol. Sci.

[287] Kayitmazer A.B., Strand S.P., Tribet C., Jaeger W., Dubin P.L. (2007). Effect of polyelectrolyte structure on protein-polyelectrolyte coacervates: Coacervates of bovine serum albumin with poly (diallyldimethylammonium chloride) *versus* chitosan. Biomacromolecules.

[288] Chang Y., McLandsborough L., McClements D.J. (2012). Cationic antimicrobial (ɛ-polylysine)-anionic polysaccharide (pectin) interactions: Influence of polymer charge on physical stability and antimicrobial efficacy. J. Agric. Food Chem.

[289] Jones O.G., McClements D.J. (2011). Recent progress in biopolymer nanoparticle and microparticle formation by heat-treating electrostatic protein-polysaccharide complexes. Adv. Colloid Interface Sci.

[290] Wandrey C., Hernandez-Barajas J., Hunkeler D. (1999). Diallyldimethylammonium chloride and its polymers. Adv. Polym. Sci.

[291] Som A., Tew G.N. (2008). Influence of lipid composition on membrane activity of antimicrobial phenylene ethynylene oligomers. J. Phys. Chem. B.

